# Effects of the tetravanadate [V_4_O_12_]^4−^ anion on the structural, magnetic, and biological properties of copper/phenanthroline complexes

**DOI:** 10.1007/s00775-023-02035-9

**Published:** 2024-01-04

**Authors:** Eduardo Sánchez-Lara, Roberto Favela, Kitze Tzian, Brian Monroy-Torres, Adriana Romo-Pérez, María Teresa Ramírez-Apan, Marcos Flores-Alamo, Antonio Rodríguez-Diéguez, Javier Cepeda, Ivan Castillo

**Affiliations:** 1 Instituto de Química, Universidad Nacional Autónoma de México, Circuito Interior, CU, 04510 Ciudad de Mexico, Mexico; 2 Facultad de Química, Universidad Nacional Autónoma de México, Circuito Exterior, CU, 04510 Ciudad de Mexico, Mexico; 3 Departamento de Química Inorgánica, Facultad de Ciencias, Universidad de Granada, Avda. Fuentenueva, 18071 Granada, Spain; 4 Departamento de Química Aplicada, Facultad de Química, Universidad del País Vasco UPV/EHU, 20018 Donostia-San Sebastian, Spain

**Keywords:** Vanadate–copper complexes, Magneto-structural correlations, Cytotoxic activity, Wound healing, Antiproliferative properties

## Abstract

The aim to access linked tetravanadate [V_4_O_12_]^4−^ anion with mixed copper(II) complexes, using *α*-amino acids and phenanthroline-derived ligands, resulted in the formation of four copper(II) complexes [Cu(dmb)(Gly)(OH_2_)]_2_[Cu(dmb)(Gly)]_2_[V_4_O_12_]·9H_2_O (**1**) [Cu(dmb)(Lys)]_2_[V_4_O_12_]·8H_2_O (**2**), [Cu(dmp)_2_][V_4_O_12_]·C_2_H_5_OH·11H_2_O (**3**), and [Cu(dmp)(Gly)Cl]·2H_2_O (**4**), where dmb = 4,4′-dimethioxy-2,2′-bipyridine; Gly = glycine; Lys = lysine; and dmp = 2,9-dimethyl-1,10-phenanthroline. The [V_4_O_12_]^4−^ anion is functionalized with mixed copper(II) units in **1** and **2**; while in **3**, it acts as a counterion of two [Cu(dmp)]^2+^ units. Compound **4** crystallized as a unit that did not incorporate the vanadium cluster. All compounds present magnetic couplings arising from Cu⋯O/Cu⋯Cu bridges. Stability studies of water-soluble **3** and **4** by UV–Vis spectroscopy in cell culture medium confirmed the robustness of **3**, while **4** appears to undergo ligand scrambling over time, resulting partially in the stable species [Cu(dmp)_2_]^+^ that was also identified by electrospray ionization mass spectrometry at *m*/*z* = 479. The in vitro cytotoxicity activity of **3** and **4** was determined in six cancer cell lines; the healthy cell line COS-7 was also included for comparative purposes. MCF-7 cells were more sensitive to compound **3** with an IC_50_ value of 12 ± 1.2 nmol. The tested compounds did not show lipid peroxidation in the TBARS assay, ruling out a mechanism of action via reactive oxygen species formation. Both compounds inhibited cell migration at 5 µM in wound-healing assays using MCF-7, PC-3, and SKLU-1 cell lines, opening a new window to study the anti-metastatic effect of mixed vanadium–copper(II) systems.

**Graphical abstract:**

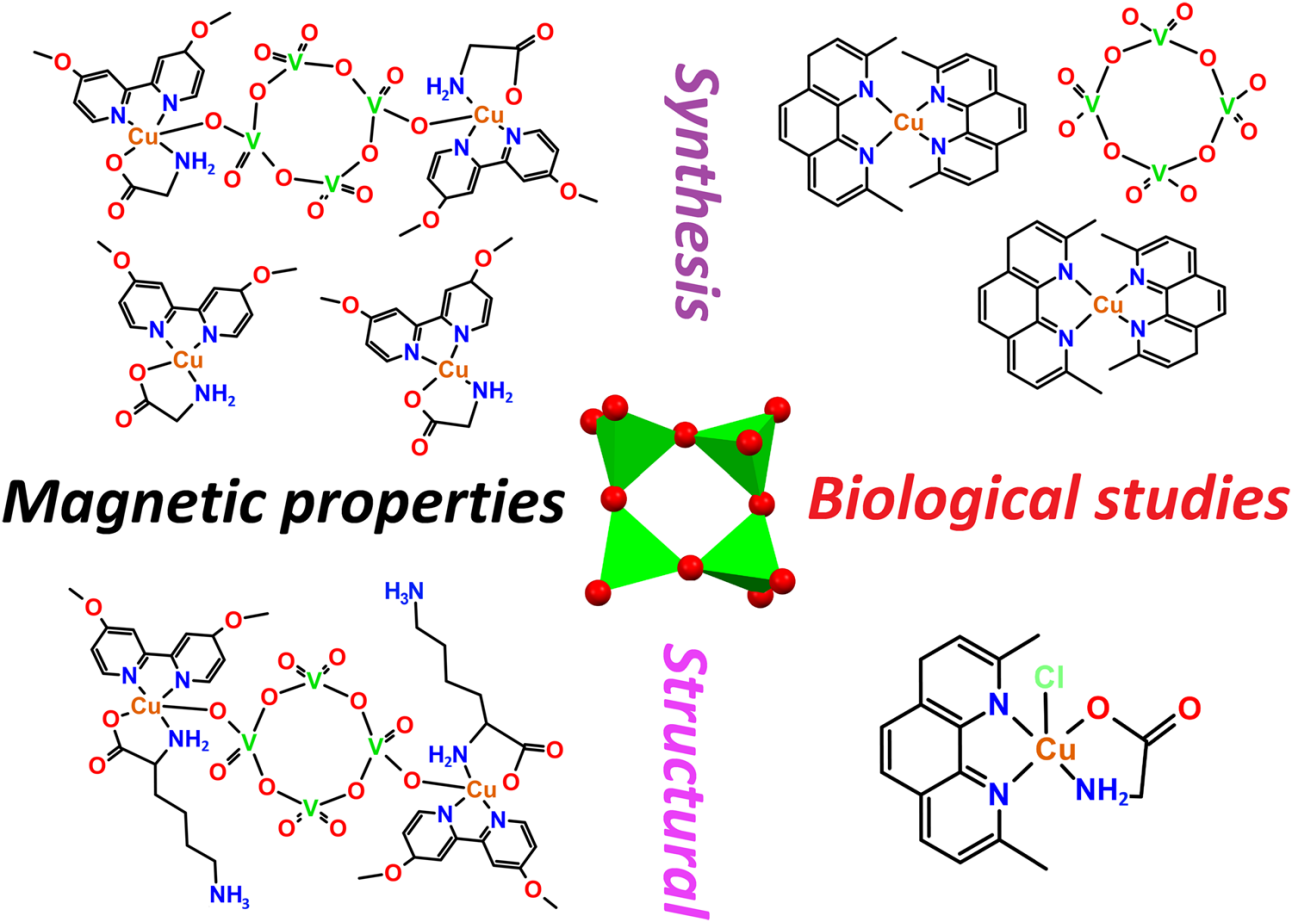

**Supplementary Information:**

The online version contains supplementary material available at 10.1007/s00775-023-02035-9.

## Introduction

Polyoxometalate chemistry is considered unique due to its unmatched versatility of properties and the wide variety of species that can self-assemble under specific experimental conditions. This is evidenced in recent reviews on this subject [1–5]. One approach that has significantly expanded the properties of these oligomeric species is their functionalization with organic or inorganic entities, making it possible to combine several properties in a single molecule [6–10]. These tailored complexes are protagonists in addressing contemporary problems with social impact regarding health, environment, energy, and information technologies [8]. Regarding human health, there has been significant research on the biological activity of polyoxometalates [11, 12]. Vanadium-based systems, including the cyclic [V_4_O_12_]^4−^ tetramer, have attracted particular attention due to their interaction with specific protein molecules and their biomedical applications, such as anticancer, antidiabetic, antiviral, and antibacterial activity, making them promising candidates for bioinorganic drugs [13, 14].

Additionally, functionalization with coordination entities has facilitated the incorporation of polyoxometalates (POMs) into magnetic materials, especially considering that the metal ions that form the constitutional units of polyoxometalates (i.e., V(V), Mo(VI), and W(VI)) often present an electronic configuration of diamagnetic nature [15–17]. Thus, decorating these species with coordination or organometallic grafts impacts their magnetic properties, allowing magnetic exchange processes and other electronic features to be tuned by modifying these scaffolds.

**Fig. 1:**
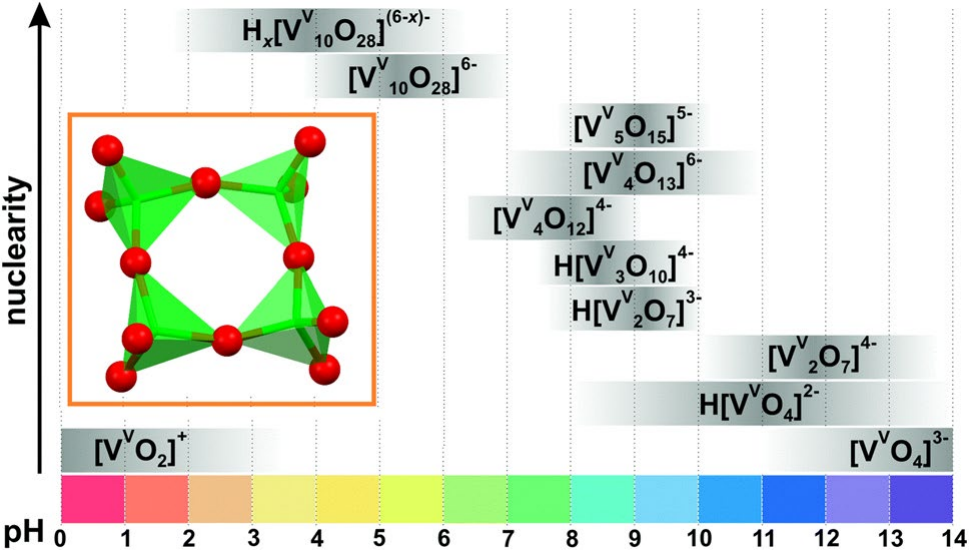
pH dependence of the nuclearity of the vanadium(V) oligomeric species. Figure reproduced with permission from Ref. [23]. Copyright 2020. Royal Society of Chemistry. The inset shows the tetravanadate [V_4_O_12_]^4−^ anion in a combined polyhedral and ball-and-stick representation

One of the essential synthetic factors when designing polyoxometalates is the pH value, which is a crucial factor involved in polyoxometalate nuclearity and its subsequent stability with counterions or other complexes [18, 19]. Another important factor in modifying the nuclearity of the POMs is the addition of heteroatoms [20], which is beyond the scope of this work. Concerning pH, tungsten and molybdenum-based POMs are stable on a predominantly acidic scale [21–23]. Polyoxovanadate chemistry is much more versatile, and oligomeric vanadium(V) species with different nuclearity can be found in a wide pH range, as confirmed by speciation studies (Fig. 1) [24–27]. Two of the most stable oligomeric vanadium species that have received particular attention are the decavanadate and tetravanadate anions. While the species derived from the [V_10_O_28_]^6−^ anion are stable at acidic pH, the *cyclo*-[V_4_O_12_]^4−^ anion is predominant at basic pH [28–31]. Their anionic nature allows solid-state stabilization using counterions from the starting materials in or intentionally adding bulky cations [32–34]. Another strategy is exploiting their anionic nature and employing cationic metal complexes to stabilize these clusters [35–38].

The present work focuses on the latter point by extending previous studies that examined the role of the tetravanadate anion as an inorganic ligand bridging copper(II) coordination units [38]. It was observed that the short Cu⋯Cu intermolecular interactions play an essential role in the magnetic properties, since the distances between these Cu^2+^ ions within the molecule is *ca.* 10 Å; furthermore, the magnetic behavior was quite different even though the components were very similar in the reported complexes. An open question that remains unanswered is the exact role of the [V_4_O_12_]^4−^ system in the magnetic behavior when it acts either as bridging or isolated counterion. In the present work, the versatility of the tetravanadate’s behavior is evidenced in the structural variations obtained, allowing us to study the last point in more detail.

A common feature in compounds that include the functionalization of the central [V_4_O_12_]^4−^ core with metal units is their low solubility, limiting their solution studies [39, 40]. This is related to the strong packing forces in the solid state favored by hydrophobic interactions of the components (observed for **1 **and **2**). The isolation of the [V_4_O_12_]^4−^ anion as a free species in **3** improved its solubility likely due to ease of solvation in polar solvents. Similarly, compound **4** that does not incorporate the polyoxovanadate anion has good solubility in water and methanol. Therefore, these two complexes were further studied in solution. This allowed us to evaluate their proliferative activity at different concentrations, and their potential as cell migration inhibitors employing the wound-healing assay in specific human tissue cancer cell lines. The role of copper(I/II) compounds with the capacity to inhibit cell proliferation has been known for years [41–43], especially those that contain planar heterocyclic motifs in their structure, such as phenanthroline derivatives, which presumably induce their therapeutic effects by DNA cleavage through an oxidative mechanism [44, 45].

Some examples of mixed copper(II) complexes featuring a heterocycle ligand and an α-amino acid exist, showing that they inhibit cell proliferation and produce dose-dependent cell death by apoptosis [46]. However, their low solubility in DMSO, a solvent unsuitable for parenteral administration at concentrations above 0.05% per day, and high toxicity have precluded their advancement as potential drugs for human use [47, 48]. As noted, complexes **3** and **4** are soluble in various organic solvents, including water for complex **4**, which is crucial in performing the preliminary biological studies we present and discuss below.

In this context, two of the three new tetravanadate-based compounds reported here with empirical formulae [Cu(dmb)(Gly)(OH_2_)]_2_[Cu(dmb)(Gly)]_2_[V_4_O_12_]·9H_2_O (**1**) [Cu(dmb)(Lys)]_2_[V_4_O_12_]·8H_2_O (**2**) (dmb = 4,4′-dimethoxy-2,2′-bipyridine, Gly = glycine, Lys = lysine) present the [V_4_O_12_]^4−^ anion in bis-monodentate bridging fashion, while in the third one with formula [Cu(dmp)_2_][V_4_O_12_]·C_2_H_5_OH·11H_2_O (**3**) (dmp = 2,9-dimethyl-1,10-phenanthroline) it acts as isolated counterion. In addition, we used a fourth mixed copper complex as a comparative model, which crystallized without the tetravanadate anion, i.e., [Cu(dmp)(Gly)Cl]·2H_2_O (**4**), allowing us to compare the solid-state effect of the polyoxovanadate ion on the structural and magnetic properties of the reported systems.

**Fig. 2:**
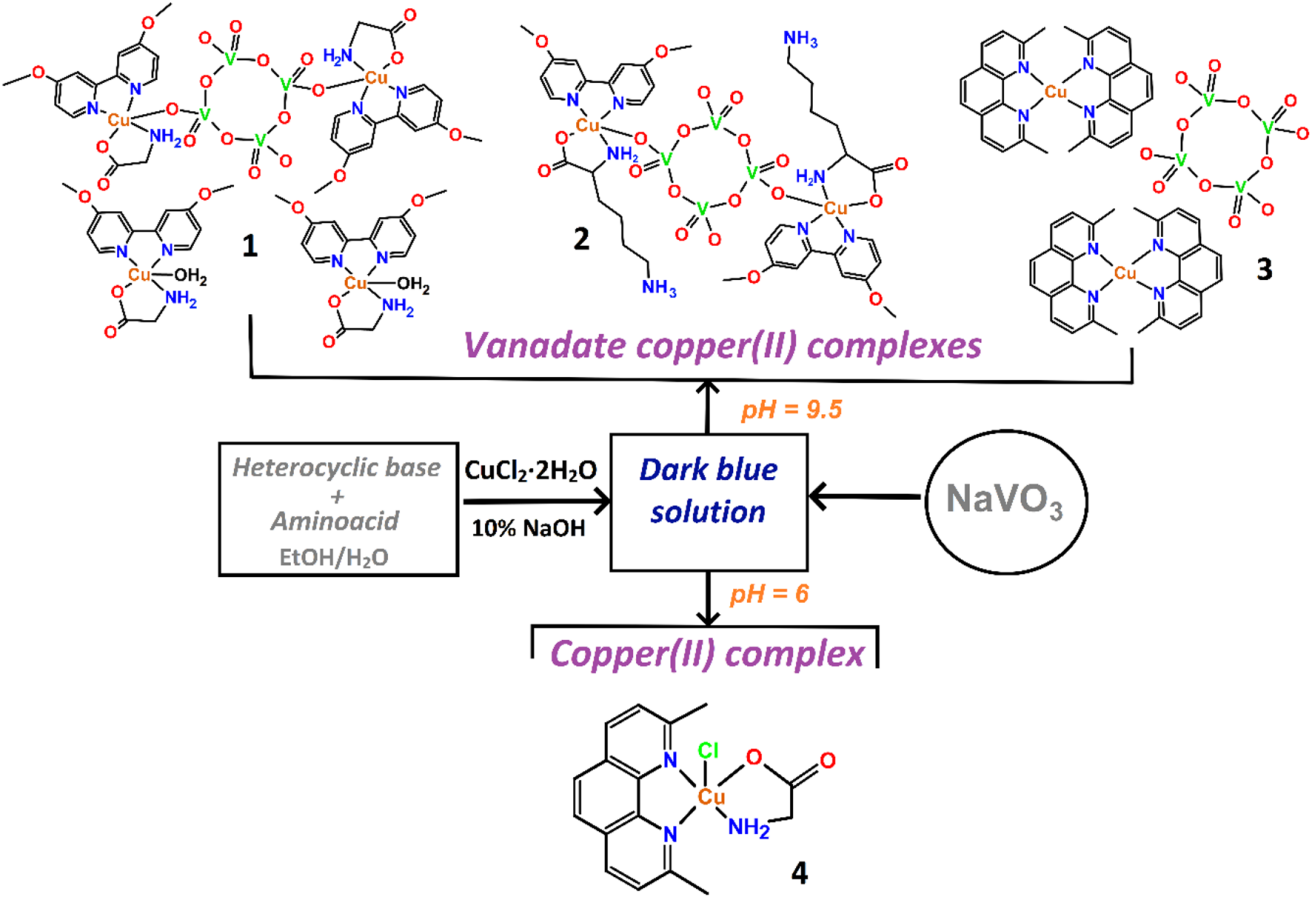
Synthetic scheme for preparation of compound **1**–**4**. For more details about the synthesis, see the text

Globally, this work presents a series of complexes that show two faces, little explored, of the tetravanadate anion. On the one hand, its role as a bridging ligand and, on the other, as a counterion. To thoroughly study this dual behavior, we have analyzed the structural, magnetic, and biological properties of the materials presented. Biological studies are focused on the cytotoxic, cell migration, and prooxidant activity of water-soluble compounds **3** and **4**. These can serve as models to understand some biochemical processes exerted by copper and vanadium ions in different biological contexts. Experimental details for obtaining all these complexes are described below and summarized in Fig. 2.

## Experimental

### General

All chemicals are of the highest commercially available purity and were used as received. Elemental analyses were obtained with a Thermo Scientific elemental analyzer Flash 2000 at a temperature of 950 °C using an XP6 Mettler Toledo microbalance; methionine from Thermo Scientific with certification number 237031 was used as standard. Mass spectra were obtained on a JEOL GC Mate II (Electron Impact) or a Bruker Esquire 600 (Electrospray Ionization/APCI-TI). EPR spectra were obtained with a Jeol JES-TE300 equipment. Electron Paramagnetic Resonance (EPR) spectra were recorded using an X-band JES-TE300 SRE Spectrometer, operated at a modulation frequency of 100 kHz with a cylindrical cavity in TE001 mode, and the spectra were acquired using the ES-IPRIT/TE program. The adjustment of the magnetic field was carried out using a JEOL ES-FC5 precision gaussmeter. Samples were analyzed in solid state at 77 K for **1**, **2**, and **3** and in a methanol solution at 77 K for **4** using quartz flat EPR cuvettes (synthetic quartz, Wilmad Glass Company) with a path length of 0.2 mm. AniSimu/FA Version 2.4.0 software was used for EPR simulation, and best fits are reported. The supplementary files included the experimental and simulated EPR spectra. Magnetic susceptibility measurements were carried out on polycrystalline samples of the complexes with a Quantum Design SQUID MPMS-7T susceptometer at an applied magnetic field of 1000 G. The susceptibility data were corrected for the diamagnetism estimated from Pascal’s Tables [49], the temperature-independent paramagnetism, and the magnetization of the sample holder.

### Preparation of complexes

Compound **1** was prepared by addition of 1.0 mmol of 4,4′-dimethoxy-2,2′-bipyridine (dmb, 0.216 g) to an aqueous solution of glycine hydrochloride (0.18 g, 1.0 mmol in 20 mL H_2_O/EtOH, 1:1 v/v) with stirring and heating to promote dissolution of dmb. Once the ligands have dissolved, the solution was cooled to room temperature. CuCl_2_·2H_2_O (0.17 g, 1.0 mmol) was then added while stirring. The pH was adjusted to 9 with the dropwise addition of NaOH (10%), giving a dark blue solution, indicating the formation of the desired copper complexes. Subsequently, NaVO_3_ (0.12 g, 1.0 mmol), previously dissolved in 10 mL H_2_O, was added to the blue solutions, favoring the immediate formation of a blue precipitate, which was collected by filtration. From the filtered solutions, blue crystals were obtained in a couple of days by slow evaporation. Compound **2** was obtained following the same synthetic procedure, replacing L-lysine hydrochloride (0. 18 g, 1.0 mmol) instead of glycine. **1:** Yield (based on vanadium): 0.115 g, 23%**.** Anal. Calc. for C_56_H_86_Cu_4_N_12_O_39_V_4_: C, 33.47; N, 8.37; H, 4.31%. Found: C, 33.05; N, 7.79; H, 4.19%; **2**: Yield (based on vanadium): 0.125 g, 35.6%. Anal. Calc. for C_36_H_66_Cu_2_N_8_O_29_V_4_: C, 30.76; N, 7.97; H, 4.73%. Found: C, 30.47; N, 7.90; H, 4.71%.

Compound **3** was obtained by a similar method, using 1.0 mmol of 2,9-dimethyl-1,10-phenanthroline (dmp, 0.216 g) added to an aqueous solution of glycine hydrochloride (0.18 g, 1.0 mmol in 20 mL H_2_O/EtOH, 1:1 v/v) with stirring and heating. CuCl_2_·2H_2_O (0.17 g 1.0 mmol) was added, and the pH adjusted to 9. To this basic solution, NaVO_3_ (0.12 g, 1.0 mmol in 15 mL H_2_O) was slowly added; no precipitate was formed. The solution was filtered to remove any trace of insoluble byproducts and left standing for slow evaporation. Red needle-shaped crystals were obtained after a few days. Yield (based on vanadium): 0.195 g, 55.7%. Anal. Calc. for C_58_H_76_Cu_2_N_8_O_18_V_2_: C, 49.68; N, 7.99; H, 5.46%. Found: C, 50.99; N, 8.20; H, 5.31%.

For compound **4,** the pH of the solution was adjusted to 6 with 10% NaOH before the addition of NaVO_3_ (0.12 g, 1.0 mmol). Adding the vanadium salt promoted the formation of a green precipitate, which was collected by filtration. Blue crystals started to deposit after 1 week. Yield (based on copper): 0.168 g, 41.73%. *Anal*. Calc. for C_16_H_20_CuClN_3_O_4_: C, 46.05; N, 10.07; H, 4.83%. Found: C, 46.22; N, 10.08; H, 4.57%.

The synthesis of these systems was performed under mild conditions using H_2_O/EtOH as solvent mixture to facilitate dissolution of the heterocyclic ligands; this is relevant since most of the reported systems with [V_4_O_12_]^4−^ have been obtained only under solvothermal conditions [50, 51]. Moreover, most crystalline systems identified using the Cambridge Crystallographic Database present complexes with homoleptic ligands. In the case of **1** and **2** that present poor solubility, crystals were obtained almost in situ by adding the vanadium precursor salt (NaVO_3_) to the basic solutions of the complexes, indicating the high stability provided by tetravanadate to the cationic Cu(II) units. High-quality single crystals with suitable size crystallize from the blue mother solutions within a few days. In the case of **3**, crystallization was slower, and due to the poor quality of the crystals, it was necessary to recrystallize them from a H_2_O/EtOH mixture. Interestingly, although the compound is soluble in ethanol, methanol, and acetonitrile, it is necessary to add water to favor and improve the crystallization process. Finally, compound **4** was synthesized similarly to **1**–**3**, except that the pH of the mixed complex was slightly acidic. Its preparation was optimized without the addition of the vanadium salt.

### Crystal structure determination

Suitable single crystals of **1**–**4** were mounted on a glass fiber and the crystallographic data were collected with an Oxford Diffraction Gemini "A" diffractometer with a CCD area detector, with *λ*_MoKα_ = 0.71073 Å at 130 K. Unit cell parameters were determined with a set of three runs of 15 frames (1° in w). The double-pass method of scanning was used to exclude any noise. The collected frames were integrated using an orientation matrix determined from the narrow frame scans. CrysAlisPro and CrysAlisRED software packages [52] were used for data collection and integration. Analysis of the integrated data did not reveal any decay. Collected data were corrected for absorption effects by an analytical, numeric absorption correction [53] using a multifaceted crystal model based on expressions upon the Laue symmetry with equivalent reflections. Structure solution and refinement were carried out with the programs SHELXS-2014 [54] and SHELXL-2014, respectively [55]. WinGX v2021 [56] and Mercury CSD 4.0 software [57] were used to prepare material for publication.

Full-matrix least-squares refinement was carried out by minimizing (*Fo*_*2*_*–Fc*_*2*_)^2^. All nonhydrogen atoms were refined anisotropically. H atoms of the water (O–H) and amine (N–H) groups were located in a difference map and refined isotropically with Uiso(H) of 1.5 Ueq and 1.2 Ueq for H–O and N–H, respectively. Hydrogen atoms attached to carbon atoms were placed in geometrically idealized positions and refined as riding on their parent atoms, with C–H = 0.95–0.99 Å with Uiso (H) = 1.2Ueq(C) for aromatic and methylene groups, and Uiso (H) = 1.5 Ueq(C) for the methyl group. For compound **2**, the atoms C11, O13, O14 and C11B, O13B, O14B are disordered over two sites with occupancies of 0.51:0.49; V3, O6, O7, O8, O10, O11 and V3B, O6B, O7B, O8B, O10B, O11B are disordered over two sites with occupancies of 0.60:0.40. Crystal data and experimental details of the structure determination are listed in Table 1. Crystallographic data have been deposited at the Cambridge Crystallographic Data Center as supplementary material CCDC: 2248349–2248352. Copies of the data can be obtained free of charge on application to CCDC, 12 Union Road, Cambridge CB2 1EZ, UK. E-mail: deposit@ccdc.cam.ac.uk.

**Table 1:** Crystallographic data and structural refinement details for **1**–**4**

	**1**	**2**	**3**	**4**
Chemical formula	C_112_H_172_Cu_8_N_24_O_78_V_8_	C_58_H_76_Cu_2_N_8_O_18_V_2_	C_58_H_76_Cu_2_N_8_O_18_V_2_	C_16_H_20_CuClN_3_O_4_
FW	4018.57	1402.25	1402.22	417.34
T(K)	130	130	130	130
*λ* (Å)	0.71073	0.71073	0.71073	0.71073
Crystal system	Triclinic	Triclinic	Triclinic	Monoclinic
Space group	$P\overline{1}$	$P\overline{1}$	$P\overline{1}$	$P2_{1} /n$
*a* (Å)	13.4073(5)	10.5096(5)	13.5951(5)	6.9366(5)
*b* (Å)	16.0172(6)	11.4461(5)	15.5897(13)	22.8465(10)
*c* (Å)	20.5849(6)	12.6380(4)	17.2372(4)	11.0420(18)
*α* (°)	89.109(3)	103.244(3)	111.935(7)	90
*β* (°)	72.735(3)	113.573(4)	108.462(5)	104.956(11)°
*γ* (°)	68.370(4)	90.052(4)	99.342(5)	90
*V* (Å^3^)	3902.1(3)	1349.03(11)	3044.3(4)	1690.6(3)
*Z*	1	1	2	4
*D*_calc._ (mg/m^3^)	1.710	1.733	1.530	1.640
Reflections collected	76,992	13,714	37,598	8249
Independent reflections	14,239 [*R*(int) = 0.0568]	2945 [*R*(int) = 0.0761]	2945 [*R*(int) = 0.0507]	3973 [*R*(int) = 0.0314]
GOF on *F*2	1.076	1.045	1.106	1.198
Final *R* indices [*I* > 2sigma(*I*)]	0.0672	0.0620	0.0546	0.0346
*wR*_2_ (all data)	0.2046	0.1377	0.1524	0.1010

### Computational details

Ab initio calculations were performed with the ORCA software suite (version ORCA 5.0.2) [58–60] to estimate magnetic parameters on X-ray models of the monomeric complexes cut from compounds **1**–**4**. All calculations were performed at the DFT level of theory with the hybrid B3LYP functional [61]. The models were completed by adding hydrogen atoms on those oxygen atoms of the [V_4_O_12_]^4−^ anion and optimizing their positions. Calculations with state-average complete active space self-consistent field (SA-CASSCF) method were performed incorporating the five d-orbitals and seven electrons (CAS(9,5) setup). Five doublet states were used to calculate these Cu(II)-based compounds. The scalar relativistic effect was considered using the zeroth‐order regular approximation (ZORA) method, for which the ZORA‐def2‐TZVPP basis set for the metal atoms and ZORA‐def2‐TZVP basis set for non-metal atoms were accordingly chosen [62–65]. NEVPT2 calculations were performed on SA-CASSCF converged wave functions to account for dynamic correlation [66]. Spin Hamiltonian parameters were also calculated from these converged results using Single_Aniso and Poly_Aniso codes as implemented in ORCA [67, 68]. On another level, the standard broken-symmetry (BS) DFT procedure using the FlipSpin feature of ORCA was followed to estimate the coupling constant value for all main superexchange pathways on dimeric models taken from X-ray coordinates.

### UV–Vis absorption spectroscopy

UV–Vis spectra of **3** and **4** in ethanol, water, and cell culture medium DMEM-F12 supplemented with 10% Fetal Bovine Serum (FBS) were measured using a Cary 100-Agilent spectrophotometer using a quartz cuvette thermostated at 300 K. The spectra were recorded between 200 and 800 nm at *t* = 0 min, 24 h, 48 h, 72 h, 96 h, and 168 h. Solutions of compounds **3** and **4** were prepared at a concentration of 50 ppm.

### Cell culture

Human cancer cell lines from different origins, including colon adenocarcinoma HCT-15, myelogenous leukemia K562, mammary adenocarcinoma MCF-7, prostate adenocarcinoma PC-3, lung adenocarcinoma SK-LU-1, glioblastoma U-251 were supplied by National Cancer Institute (USA), and the Cancer Institute of Mexico donated one healthy monkey kidney cell line COS-7. Cells were cultured in RPMI-1640 medium (Gibco^®^) supplemented with fetal bovine serum (10% v/v), nonessential Amino Acids (1% v/v), and penicillin streptomycin solution (1% v/v) (Corning^®^). Cells were maintained at 37 °C in a humidified atmosphere with 5% CO_2_.

### Cytotoxic activity

Cytotoxicity of **3** and **4** was tested against COS-7, HCT-15, K562 MCF-7, PC-3, SK-LU-1, and U-251 cell lines by the sulforhodamine B (SRB) assay. Cells were seeded in a 96-well plate and incubated with compounds for 48 h, after cell monolayers were fixed by adding cold trichloroacetic acid (50% wt/v) to each well directly to the medium supernatant and incubated for 1 h at 4 °C, after that time the plates were gently washed with water and dried at room temperature. To cell dye, 100 μL of SRB (0.4% wt/v) was added to each well, left at room temperature for 30 min, and then washed with acetic acid (1% v/v) to remove the unbound dye and dried at room conditions. Protein-bound was solubilized by adding 100 μL tris base solution (10 mM) and shaking on an orbital shaker for 10 min. The absorbance was obtained at 515 nm in a microplate reader (SYNERGY HT, BioTek). The dose–response curve was plotted for each most active compound and the IC_50_ was estimated from linear regression. The results express the mean IC_50_ ± SEM obtained from three independent experiments performed at 48 h.

### Lipid peroxidation experiments

#### Animals

Adult male Wistar rats (200–250 g) were provided by the Instituto de Fisiología Celular, UNAM. Adult male Wistar rats were approved by the Animal Care and Use Committee (NOM-062-ZOO-1999). They were maintained at 25 °C on a 12/12-h light–dark cycle with free access to food and water.

#### Rat brain homogenate preparation

The animal euthanasia was carried out avoiding unnecessary pain. Rats were euthanized with CO_2_. The cerebral tissue (whole brain) was rapidly dissected and homogenized in phosphate buffered saline (PBS) solution (0.2 g KCl, 0.2 g KH2PO_4_, 8 g NaCl and 2.16 g NaHPO_4_·7H_2_O L^−1^, pH adjusted to 7.4), as reported elsewhere to produce a 1/10 (w/v) homogenate [69]. Then, the homogenate was centrifuged for 10 min at 1100g (*ca.* 3400 r.p.m.) to yield a pellet that was discarded.

#### Protein content in brain supernatant solutions

The protein content in brain supernatant solutions was measured using the Folin and Ciocalteu’s phenol reagent [70] and adjusted to 2.66 mg protein mL^−1^ with PBS solution.

#### Induced lipid peroxidation

All experiments were conducted in ice bath. Supernatant solution (375 µL, 2.66 mg protein mL^_1^) were added together with 50 µL of 20 µM EDTA and 50 µL of PBS solution to microtubes. The brain contains high levels of Fe that induce lipid peroxidation (LP) by its own right [71]. Adding EDTA to all samples served the purpose of chelating iron originally present in the brain homogenates. Then, 25 µL aliquots of compounds in 50% methanol were added to microtubes. Final concentrations of test compounds are reported in supporting material (Fig. S20). Final concentration of protein and EDTA were 2 mg mL^−1^ and 2 µM, respectively. Then, the mixture was incubated at 37 °C for 3 h in a Lab Line Titer Plate Shaker Model 4265 at 1.5 constant shaking speed.

#### Thiobarbituric acid reactive substances (TBARS) quantification

A modified method described elsewhere for TBARS quantification was used [72]. The method is based on the reaction of malondialdehyde (MDA), a product of LP [73], and other TBARS (substances formed in the course of the reaction that reacting with TBA give an adduct whose spectrum is identical with that obtained from MDA standard [74] with TBA in a 1:2 M ratio on heating to give a red adduct whose concentration is related to the extent of LP [74]. A 1% (w/v) TBA solution in 0.05 N NaOH was prepared and mixed with 30% (w/v) TCA in 1:1 proportion. A half-mL aliquot of the TBA reagent was added to each microtube. The tubes were cooled on ice for 10 min, centrifuged at 13400 g for 5 min, and finally heated at 95 °C for 30 min. The tubes were allowed to reach ambient temperature. Two-hundred microliters aliquots of the supernatant solution were separated for analysis. The content of TBARS in the suspensions was determined by optical density at *λ* = 540 nm using a Bio-Tek Synergy HT Microplate Reader. The concentrations of TBARS were calculated by interpolation from an experimental standard curve determined for tetramethoxypropane (TMP) as described elsewhere [75]. All data were represented as mean ± standard error. One-way ANOVA followed by Dunnett’s test for comparisons against control were conducted for data analyses.

#### Wound healing assay

To check the effect of **3** and **4** on cancer cell migration, wound-healing assay was performed in MCF-7, PC-3, and SK-LU-1 cell lines. The cells were seeded in 24-well plates at a density of 2 × 10^5^, 1 × 10^5^, and 2.1 × 10^5^ cells per well, respectively, and then cultured for 48 h (38 °C and 5% CO_2_) to reach 90–100% confluence. An artificial space was generated in the cell monolayer using a 200 μL micropipette tip. Floating cells were removed by washing with PBS. Subsequently, the cells were cultured with a new medium supplemented in the absence and presence of the copper complexes at a concentration of 5 μM, using mitoxantrone (MTX) as a positive control at 0.5 μM. Images were captured by an inverted microscope (DIAPHOT 300 Nikon^®^, Japan) with a digital camera (AmScope MD500) at 0, 24, and 48 h of treatment. Wound areas were obtained using ImageJ software's polygon selection and 'Measure' tool. The relative migration rate (%) was calculated using the following equation:

$${\text{Relative migration ratio}}\,\left( \% \right) = \frac{{\left( {{\text{wound area}}\,0\,{\text{h}} - {\text{wound area}}\,24\,{\text{or}}\,48\,{\text{h}}} \right)}}{{{\text{wound area}}\,0\,{\text{h}}}} \times 100.$$

Experiments were carried out in triplicate, and a two-way ANOVA obtained significance with Tukey’s multiple comparisons tests.

## Results and discussion

### Synthesis strategy

Self-assembly of polyoxovanadates relies on the pH as one of the most critical parameters to consider during the synthetic process [19, 34, 76]. For **1**–**3**, the pH chosen was basic (~ 9) to obtain the cyclic tetravanadate [V_4_O_12_]^4−^ anion, which is stable at pH > 8. At neutral or acidic pH values, the predominant polyoxovanadate is the orange-colored decavanadate [V_10_O_28_]^6−^ cluster [23, 24, 77, 78]. However, considering the nature of the ligands used in this work, it is more feasible to stabilize [V_4_O_12_]^4−^ to avoid protonation of the amino acid co-ligands. 4,4′-dimethoxy-2,2′-bipyridine (dmb) was used in the synthesis of **1** and **2**, while 2,9-dimethyl-1,10-phenanthroline (dmp) was employed for **3**. These neutral ligands stabilize copper(II) complexes in a wide pH range forming stable 5-membered chelate rings [79, 80]. The essential amino acids used in this work to form the mixed complexes are glycine and lysine, which behave differently at basic pH due to their acid–base properties, a feature exploited in this work.

In this context, the isoelectric point (*IP*) of glycine is 5.96 [81], so at basic pH the *α*-carboxylic group is deprotonated (COOH → COO^−^) and can be accessible to the Cu^2+^ ion, contributing with a negative charge to the complex as a glycinate, as indeed occurs in **1**. Despite working at a strongly basic pH value (around 9), we did not observe the formation of homoleptic copper glycinate [Cu^II^(Gly)_2_], revealing that the use of dmb as co-ligand is also a key factor for the design of the mixed complex [Cu(dmb)(Gly)]^+^ (see Fig. 2). Lysine has an *IP* of 10.53 [82], making it an ideal bidentate ligand for metal ions at basic pH because the amino acid exists as a zwitterion; in other words, the *α*-carboxylic group is deprotonated—accessible to Cu^2+^ ion—and the amino group of the side chain is protonated. In this configuration, the molecule does not contribute with any charge to the coordination moiety, affording [Cu(dmb)(Lys)]^2+^. Thus, the basic pH facilitated the formation of the tetravanadate [V_4_O_12_]^4−^ cluster from the precursor NaVO_3_, and simultaneously the formation of the copper(II) chelates in **1**–**3**.

Replacement of dmb with dmp to get the mixed copper(II) complexes with glycine or lysine leads instead to **3**, where the tetravanadate [V_4_O_12_]^4−^ anion behaves as a non-coordinating counterion of two homoleptic [Cu(dmp)_2_]^2+^ units (Fig. 2). Efforts to get the mixed-ligand complexes with the corresponding amino acids at basic pH were unsuccessful, indicating that dmp enforces formation of homoleptic copper(II) entities [83, 84]. The bulky methyl groups in *ortho* position to the nitrogen atoms of dmp likely prevent coordination of the metal center to the [V_4_O_12_]^4−^ anion observed in **1** and **2**, promoting the stability of the complex salt.

Considering these results, we conducted the reaction at a slightly acidic pH (around 6), expecting that other vanadium oligomeric species, such as the [V_10_O_28_]^6−^ anion, would be formed in the presence of dmp, thus affording variation of polyoxovanadate cluster. Instead, this approach favored the formation of blue single crystals that revealed a structure corresponding to a mixed-ligand copper complex containing no vanadium-containing counterion upon analysis. Furthermore, the complex obtained (**4**) allowed us to compare its properties with those of tetravanadate-containing systems.

### Crystal structure description

**Fig. 3:**
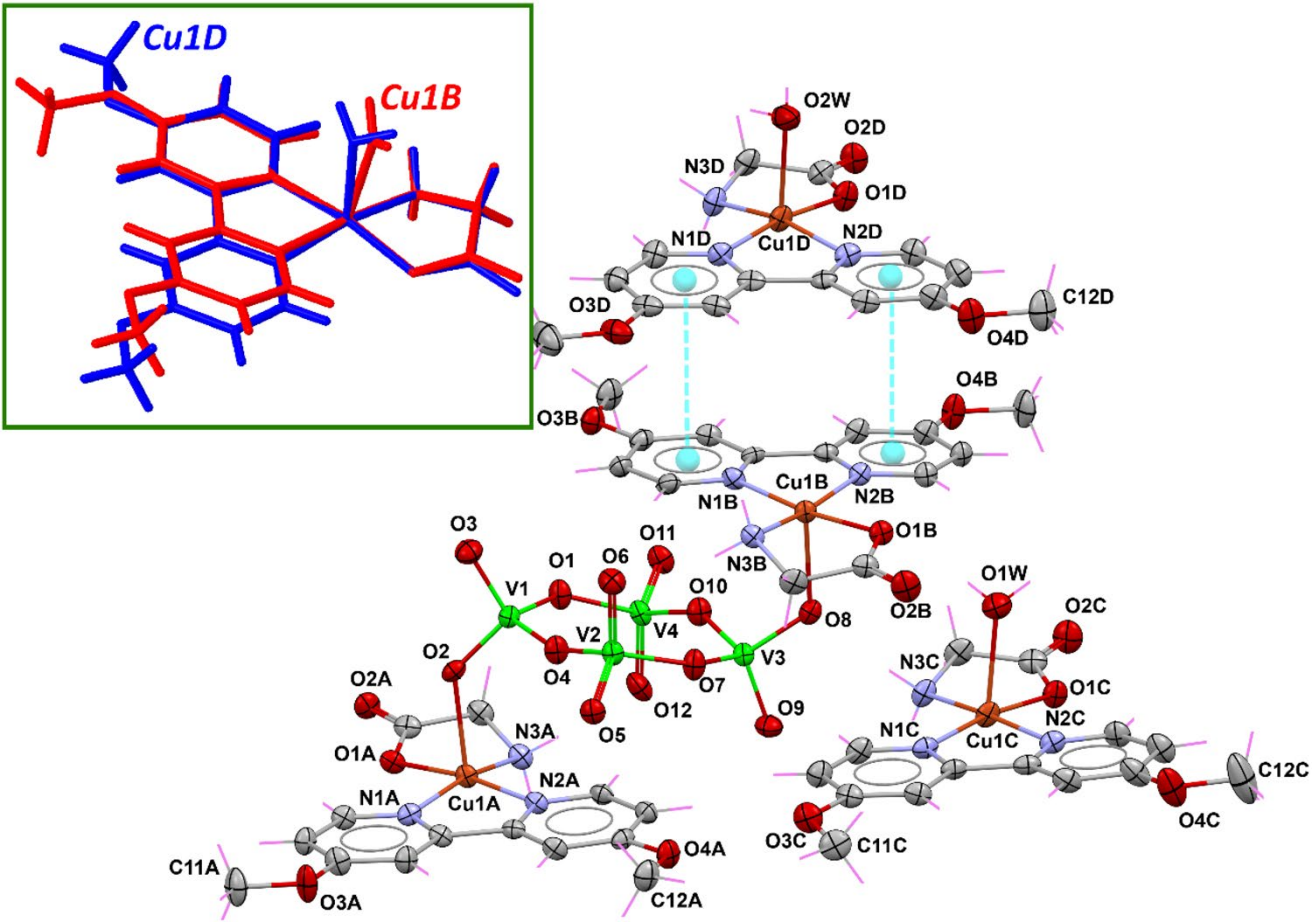
Asymmetric unit of **1** with displacement ellipsoids for non-H atoms at 50% probability level. The inset shows the overlay between Cu1B and Cu1D entities

Compound **1** crystallized in the triclinic space group $P\overline{1}$ (Table 1) with three copper(II) complexes in the asymmetric unit, i.e., two complex cations [Cu(dmbp)(Gly)(H_2_O)]^+^ with a similar structural pattern and a heterobimetallic Cu-V coordination compound {[Cu(dmb)(Gly)]_2_[V_4_O_12_]}^2−^ (Fig. 3). This arrangement can be rationalized from detailed analysis of its structural features.

In the cations present in the asymmetric unit, the glycinate anion stabilizes the Cu(II) ions (Cu1A and Cu1B) with a negative charge via the deprotonated COO^*−*^ group. In contrast, the dmb molecules are coordinated in a neutral fashion to the same metal ion. These two ligands occupy the basal plane of the square pyramidal (SPY) environment around Cu1A and Cu1B, whereas a tetravanadate oxygen atom occupies the apical position. A detailed environmental analysis using continuous shape measurements (CShMs) with the SHAPE program [85] confirms the low distortion concerning the reference polyhedron (SSPY = 0.984 and 0.981, see Table S1). Therefore, the two Cu(II) complexes linked to the tetravanadate [V_4_O_12_]^4−^ cluster stabilize this group with a positive charge each, forming the anionic entity {[Cu(dmbp)(Gly)]_2_[V_4_O_12_]}^2−^. Interestingly, this unit is electrostatically stabilized in the lattice by two discrete copper complexes [Cu(dmbp)(Gly)(H_2_O)]^+^, generating the complete neutral asymmetric unit. A set of solvent water molecules placed in general positions complete the molecular formula of **1**, {[Cu(dmbp)(Gly)]_2_[V_4_O_12_][Cu(dmbp)(Gly)(H_2_O)]_2_}·10H_2_O.

In both Cu1C and Cu1D, the metal ions are bound to the chelating dmb ligand, the glycinato anion through the *α*-amino and carboxylate functional groups, and to one coordinating water molecule. In Cu1D, where the square pyramidal base is defined by N1D/N2D/N3D/O1D atoms, the copper ion is displaced 0.136 Å out of the mean plane formed by these basal atoms, with bond lengths in the range of 1.960(4)–2.010(5) Å. For the Cu1C unit, the copper–ligand distances lie in the range of 1.968(5)–2.010(5) Å and Cu1C is situated slightly above the N1C/N2C/N3C/O1C plane with a distance of 0.078 Å. In both complexes, a coordinating water molecule defines the apical position with Cu–O_w_ distances of 2.311(5) and 2.281(5) Å for Cu1C and Cu1D, respectively. Table S2 summarizes the distances around copper(II) ions for all compounds.

A detailed analysis with SHAPE program (Table S1) reveals that probably due to the longer Cu–Ow distance, the environment around Cu1C is best described as a vacant octahedron (vOC) in terms of its less distortion concerning that reference polyhedron (S_vOC_ = 1.121 vs. S_SPY_ = 1.193). At the same time, Cu1D better fits a square pyramid (SPY) environment (S_SPY_ = 0.889). The square pyramidal coordination environments have an angular structural parameter (*τ*_5_) of 0.061 for Cu1C and 0.081 for Cu1D, reflecting a limited distortion toward the trigonal–bipyramidal geometry [86]. In short, and as shown in the inset of Fig. 3, these cationic complexes have very similar structural details.

The heterometallic molecule comprises the linkage to the [V_4_O_12_]^4−^ ion with the paramagnetic [Cu(dmbp)(Gly)]^+^ units to yield [Cu(dmbp)(Gly)]_2_[V_4_O_12_]. The polyoxovanadate comprises four vertex-sharing tetrahedral {VO_4_} units, generating a cyclic motif with a chair-type conformation [34, 87]. The vanadium atoms and the bridging oxygen form a square-like {V_4_O_4_} ring, as represented in the inset of Fig. 1. Comparison of [Cu(dmbp)(Gly)]_2_[V_4_O_12_] with the universe of known inorganic crystal structures is most easily performed by reading the CIF into *Mercury* and executing the *CSD System/Mogul Geometry Check* [88, 89]. In this case, the bond distances, angles, and all of the torsion angles of the tetravanadate were *not unusual (enough hits)*, indicating that they fall within the customarily expected ranges. On the other hand, the O atoms from the bridging [V_4_O_12_]^4−^ unit define the apical positions of the [Cu(dmbp)(Gly)]^+^ entities with Cu1A–O2 and Cu1B–O8 distances of 2.267(4) and 2.269(4) Å, respectively.

In the crystal structure, the [Cu(dmbp)(Gly)]_2_[V_4_O_12_] complexes are packed such that intermolecular interactions are favored between the metal ions and the O atoms from adjacent COO^*−*^ groups with Cu1A⋯O2B and Cu1B⋯O2A distances of 3.080 and 3.175 Å, respectively (Fig. S1). These contacts are common when the Jahn–Teller effect prevents octahedral symmetry, avoiding the formation of coordination polymer structures but allowing contacts between the carboxylate oxygen donor and the metal centers [34]. These interactions occur between symmetry-related complexes stacked along the *a*-axis and bring about infinite supramolecular {[Cu(dmbp)(Gly)]_2_[V_4_O_12_]}_n_ chains that can give rise to magnetic interactions: relatively short Cu1A⋯Cu1B distances of *ca.* 5.10 Å are established along the pathway derived from this packing scheme (Fig. S1, inset).

**Fig. 4:**
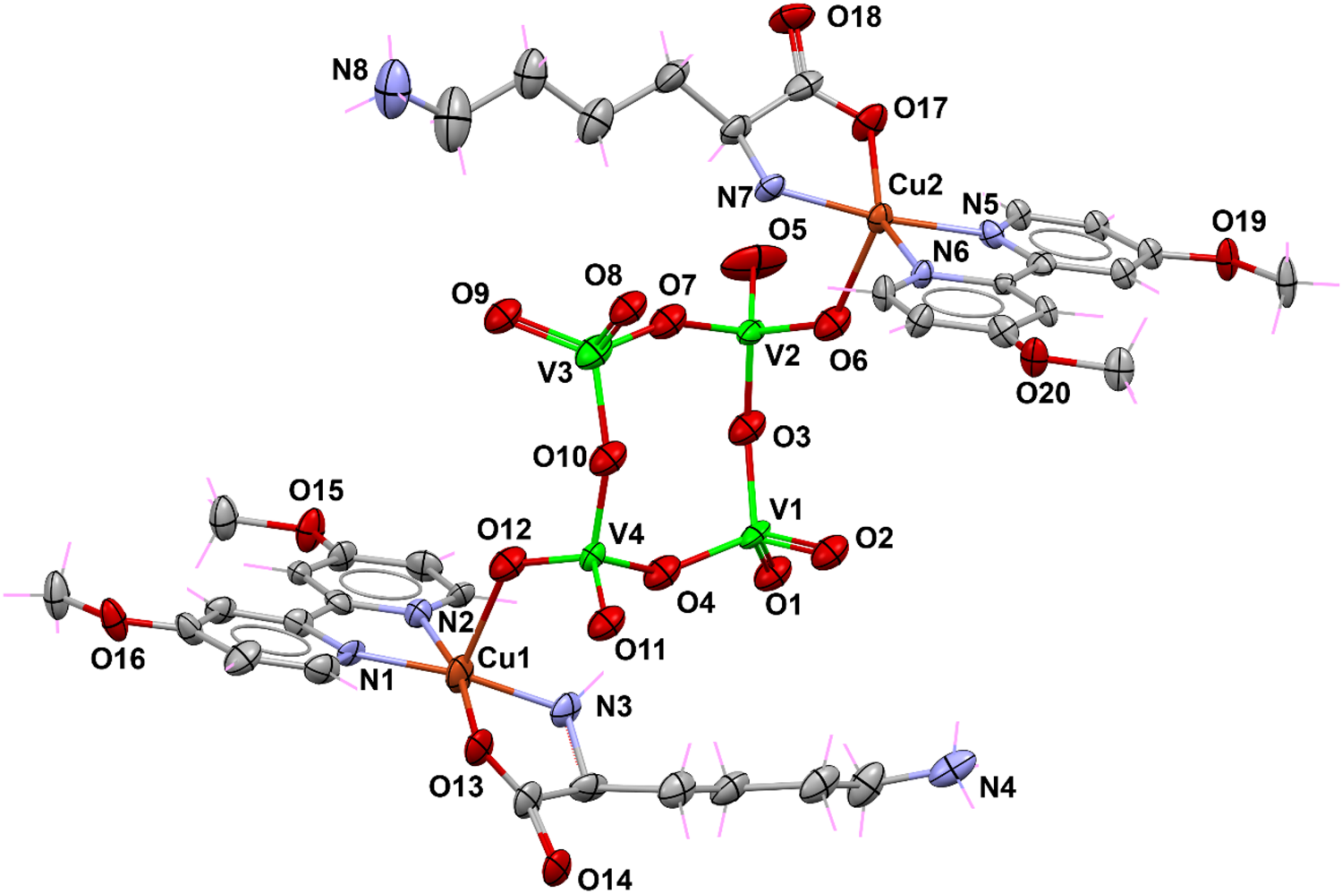
Crystal structure of **2** with displacement ellipsoids for non-H atoms at 50% probability level. Water molecules are omitted for clarity

Solid-state characterization of **2** (Fig. 4) provides insight into the structural differences that arise when a different amino acid is used, keeping the dmb ligand intact. Lysine has two acid–base (titratable) groups [82], and its zwitterionic nature results in significant structural effects. Compound **2** crystallizes in the enantiomorphic *P*1 space group (Table 1). As in **1**, the asymmetric unit matches its molecular formula, which consists of [Cu(dmb)(Lys)]_2_[V_4_O_12_]·8.5H_2_O, where the [V_4_O_12_]^4−^ anion serves as a bridge between two [Cu(dmb)(Lys)]^2+^ coordination units. The two crystallographically independent copper(II) ions balance the charge of the vanadium system.

In this crystal structure, copper(II) ions bound to polyoxovanadate exhibit a distorted square pyramidal geometry as confirmed by CShMs, with S_SPY_ = 0.892 and 1.447 for Cu1 and Cu2 (Table S1), with the N donors of dmp and the N and O atoms of the *α*-amino acid coordinated in the basal plane. A tetravanadate O atom defines the apical position of both metal ions (Fig. 4). The basal distances differ slightly from **1**, ranging from 1.926(15) to 2.001(8) for Cu1, and 1.947(7) to 2.007(9) Å for Cu2 (Table S2). Moreover, the distortion around the copper ions is more pronounced than in **1** due to a structural effect of the side chain of the lysine molecule and to the occupational disorder of the COO^*−*^ group around Cu1. Regarding the apical positions, the Cu–O distances are 2.306(8) Å for Cu1–O12 and 2.223(13) Å for Cu2–O6. Again, the copper ions interact with the O atoms from the carboxylate group of an adjacent molecule in the crystal lattice, which can potentially contribute to the magnetic behavior.

The molecules of [Cu(dmbp)(Lys)]_2_[V_4_O_12_] are further held through *π*–*π* stacking interactions between the aromatic rings of dmb, forming layers parallel to the *bc* plane. The centroid–centroid distances between these systems are 3.460 Å for N1/N6 and 3.497 Å for N2/N5. This arrangement gives rise to two intermolecular Cu⋯O interactions with distances of 3.178 Å and 3.449 Å for Cu1⋯O18 and Cu2⋯O14, respectively (see expanded view of Fig. S2). In this scheme, the Cu1⋯Cu2 separation is 5.180 Å, of the same order as that observed in **1** in such a way that similar magnetic behavior may be expected.

**Fig. 5:**
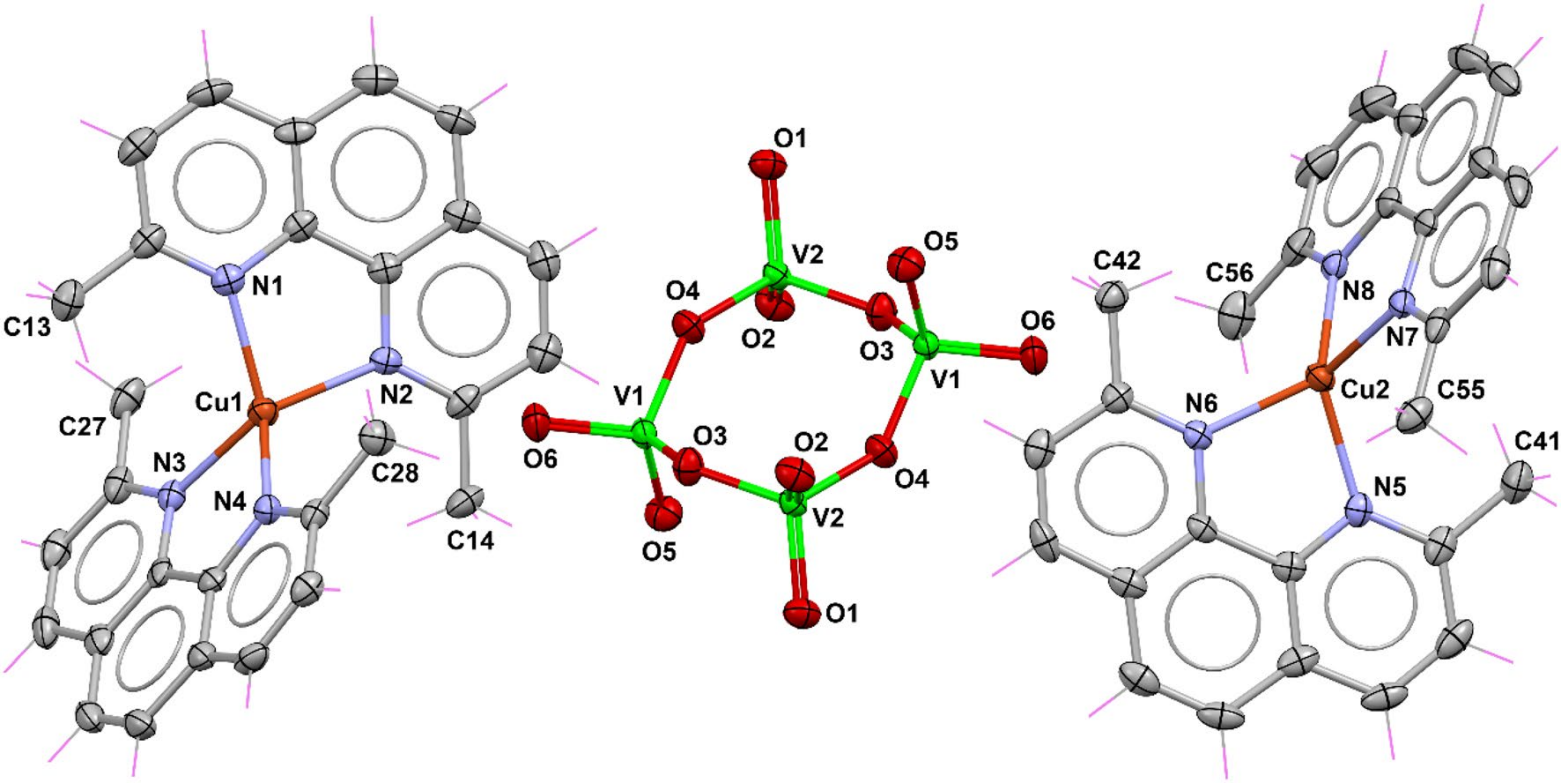
Part of the crystal structure of **3** with displacement ellipsoids for non-H atoms at 50% probability level. Water molecules are omitted for clarity

Compound **3** crystallizes in the triclinic space group $P\overline{1}$, with a high content of crystallization water molecules. Unlike **1** and **2**, where [V_4_O_12_]^4−^ acts as a monodentate ligand, in **3** it acts as a counterion to balance the positive charge of two symmetrically non-equivalent copper(II) homoleptic [Cu(dmp)_2_]^2+^ complexes (Fig. 5). According to the geometry index *τ*_4_ introduced by Yang et al. [90] as an extension of the *τ*_5_ parameter, values of 0.65 and 0.67 are obtained for Cu1 and Cu2, respectively—for a perfect tetrahedral geometry *τ*_4_ equals 1.00; while for an ideal square planar geometry, it corresponds to 0—indicating that the coordination environment around the copper ions consists of a distorted tetrahedral geometry. CShMs confirm this (Table S3), with the closest reference polyhedron for the metal centers being a tetrahedron (Td), although both are highly distorted (*S*_Td_ = 6.480 and 6.678, respectively, for Cu1 and Cu2).

The geometric constraints imposed by the methyl substituents of the neocuproine backbone prevent the planar geometry. In particular, the dihedral angles between the planes defined by the copper center and each set of nitrogen atoms of the neocuproine ligands are 71.95(11)° for Cu1 and 75.00(12)° for Cu2. These values differ from the dihedral angles of 58.45° and 62.38° measured for the previously reported complexes [Cu^II^(dmp)_2_](ClO_4_)_2_ and [Cu^II^(dmp)_2_](BF_4_)_2_, respectively [91, 92]. On the other hand, the Cu–N distances fall in the range of 2.032(3) Å–2.050(3) Å for Cu1 and 2.047(3) Å–2.062(3) Å for Cu2 (Table S2), which are comparable to those observed for [Cu^II^(dmp)_2_][CF_3_CO_2_], [Cu^II^(dmp)_2_][BF_4_]_2_ and [Cu^II^(dmp)_2_][ClO_4_]_2_ [93].

The dicationic [Cu(dmp)_2_]^2+^ has been crystallized with a series of counterions; however, according to a CSD search for substructures containing this cation, there are only three reported compounds forming salts with this complex cation, having been co-crystallized with the anions ClO_4_^−^, BF_4_^−^, and CF_3_CO_2_^−^ [91–93]. Six more structures contain a solvent molecule (i.e., MeCN, H_2_O, or MeOH) incorporated into the coordination sphere of pentacoordinate complexes [83, 94, 95]. In contrast, there are 58 structures with the [Cu(dmp)_2_]^+^ monocation, indicating that the neocuproine ligand stabilizes copper in a low oxidation state [83, 96–99]. In the case of **3**, this reduction may be hampered by the high stability conferred by the [V_4_O_12_]^4−^ anion to the [Cu(dmp)_2_]^2+^ units.

Crystal packing results in symmetrically non-equivalent [Cu(dmp)_2_]^2+^ stacked and interacting through π–π contacts between two central rings of the neocuproine ligands with equal distances of 3.679 Å (Fig. S3). In this arrangement, the intermolecular Cu1⋯Cu2 distance is 6.76(7) Å, which may be considered very large to allow a strong magnetic interaction.

**Fig. 6:**
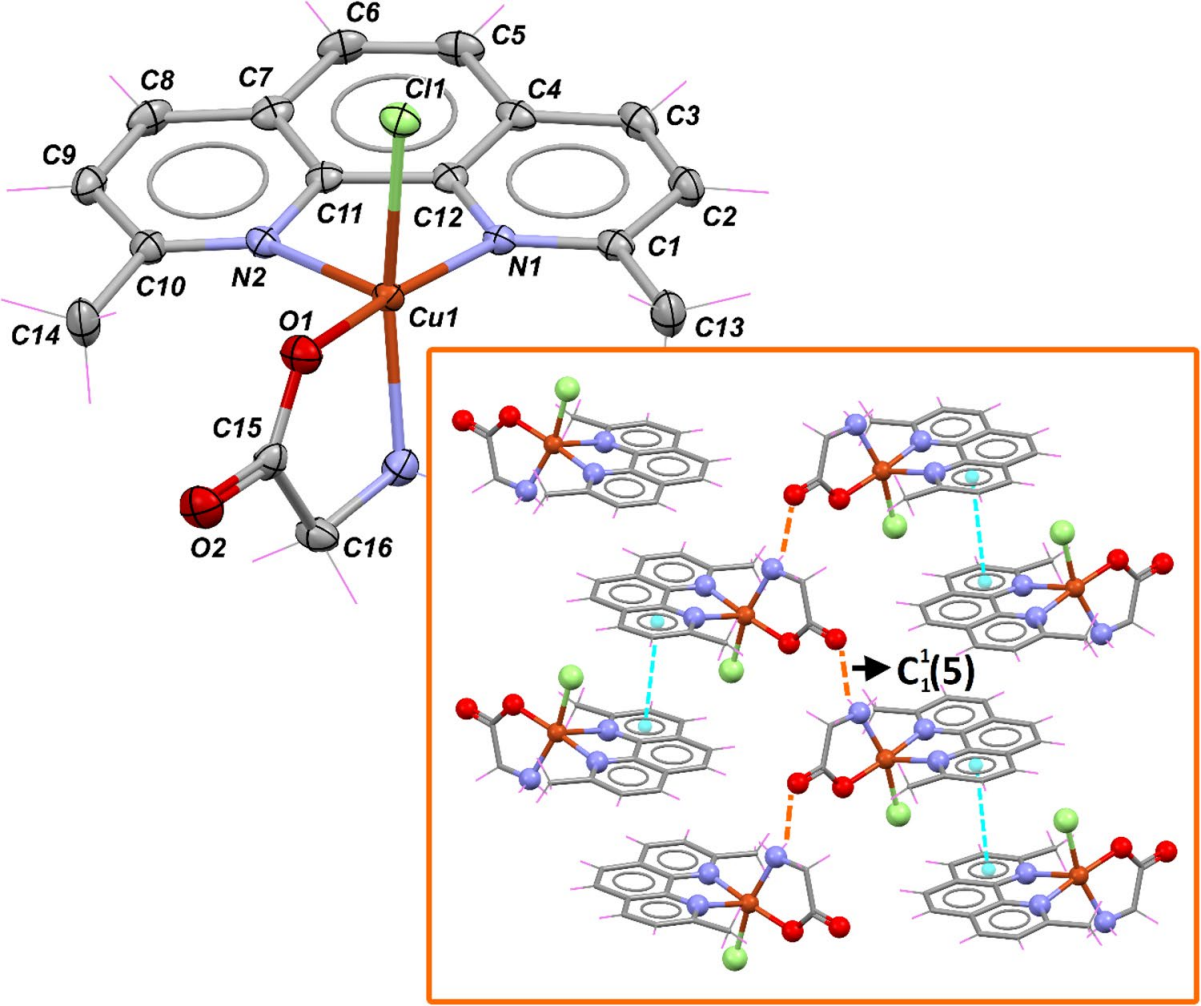
Crystal structure of **4** with displacement ellipsoids for non-H atoms at 50% probability level. The inset shows the packing of the molecules in the lattice, highlighting the stacking between the heterocyclic ligands (magenta dashed lines) and the NH⋯O hydrogen bonds (orange dashed lines)

Finally, compound **4** crystallizes in the monoclinic space group $P2_{1} /n$ with four complexes in the unit cell. According to the value of *τ*_5_ = 0.13 [86] calculated for 5-coordinate structures, the geometry around copper(II) best fits a distorted square-based pyramid in which the metal ion is coordinated to the nitrogen atoms of dmp, and N,O-donor sets of the glycinate anion in basal positions, while a chloride ion completes the apical position (Fig. 6). CShMs support the previous conclusion by showing the least distortion concerning SPY (S_SPY_ = 1.904; Table S1). The Cu–N and Cu–O distances are in the range of 2.014(2)–2.271(2) and 1.9530(19), respectively, while the Cu–Cl distance is elongated with a distance of 2.309(7) Å.

It is quite possible that the glycinate ion's coordination mode and the chloride ion's presence in the inner coordination sphere confer stability to **4**, preventing the incorporation of the [V_4_O_12_]_4-_/[V_10_O_28_]^6−^ species into the crystal lattice. Reviewing the supramolecular structure with the *graph-set* [100] menu available in *Mercury,* the presence of chains between two complexes with an intermolecular N–H⋯O distance of 2.83 Å that extends along the crystallographic *c*-axis is evident. The dmp molecules are packed through *π*–*π* interactions with a centroid–centroid distance of 3.561 Å between two symmetry-related [Cu(dmp)(Gly)Cl] systems (Fig. 6, inset). The Cu⋯Cu distances are longer than in the previous compounds due to the presence of voids filled by lattice water molecules, which push away the complex molecules. In this sense, the shortest Cu⋯Cu distance is 8.66 Å, making any magnetic interaction unlikely.

### Vibrational spectroscopy

The vibrational spectra of complexes **1**–**4** are shown in Fig. S4. Generally, the bands in the high-frequency region (3400‒2900 cm^−1^) are not metal-sensitive since they originate in the heterocyclic or aromatic ring of the organic backbones. Therefore, the IR spectra description focuses on the low-frequency region, where *ν*(M‒N) and other metal-sensitive vibrations appear [101]. Thus, we can observe certain similarities in the spectra of **1**–**3** from 1000 to 400 cm^−1^ that correspond to intense *ν*(V–O) vibrations of the cyclo-[V_4_O_12_]^4−^ anion with overlapping ligand vibrations in the same frequency region [102]. Likewise, the IR spectra of **1 **and **2** are very similar in the region of 1600‒1200 cm^−1^ since the heterocyclic ligand is the same for both complexes; this is also the case for the asymmetric and symmetric vibrations of the COO^‒^ moiety from the corresponding amino acids.

The coordination mode of carboxylates can be inferred from the separation of the bands placed at different frequencies (i.e., Δ*ν* = CO_as_‒CO_s_) relative to those of the free carboxylate ion [101]. In this context, the glycinate ion in **1** exhibits *ν*_as_(COO^‒^) and *ν*_s_(COO^‒^) at 1607 cm^−1^ and 1390 cm^−1^, respectively (Δ*ν* = 217), while the lysinate donor in **2** displays these vibrations at 1605 cm^−1^ and 1395 cm^−1^, respectively (Δ*ν* = 210). These values correlate with a monodentate coordination fashion by the carboxylate group, which agrees with the X-ray diffraction data for both complexes. On the other hand, the 1660–990 cm^−1^ frequency region in **3** is quite different from that of compounds **1** and **2** due to the neocuproine presence and the missing amino acid [103, 104]. This spectral region is mainly associated with the stretching vibrations of dmp *ν*(C=C) and *ν*(C=N) around 1500–1400 cm^−1^ and with the bending vibrations of the *δ*(C‒H) in the region of 1400–1000 cm^−1^.

Naturally, for **4** we did not observe the intense stretching and bending vibrations typical for oligomeric vanadium(V) species. However, besides serving as a model to differentiate the vanadate vibrations from the ligands forming the complexes in the low-frequency region, we can also visually compare the organic scaffolds vibrations of this compound with the compounds **1**–**3** in the 1600–1000 cm^−1^ region and draw some conclusions. IR spectroscopy is insufficient to distinguish the tetravanadate binding mode in the free species, undoubtedly a consequence of the high rigidity of the anion and packing effects.

### EPR spectroscopy

The g_⊥_ and g_‖_ values observed in the EPR analysis of **1**–**4** are within the expected ranges considering their coordination environments (Table 2) [29, 105, 106]. Compound **1** shows an EPR spectrum with axial symmetry, which is expected for copper(II) species with a square-based pyramidal geometry (Fig. S5a), where g_‖_ > g_⊥_ and a *d*_*x*_^2^_─*y*_^2^ ground state. However, an important feature observed is the absence of the hyperfine interaction of the metal ion (*A*). This behavior can be attributed to possible dipole–dipole interactions between the molecules in the solid state and the spin relaxation rate [106–108]. This results in a broadened signal without the typical hyperfine splitting. Considering the short Cu⋯Cu intermolecular interactions observed in the crystal packing of **1**, the geometrical parameter (G) was calculated to determine the exchange interaction between these metal centers through the following equation:

$$G = \left( {g_{\parallel } - 2.0023} \right)/\left( {g_{ \bot } - 2.0023} \right).$$

**Table 2:** Experimental and simulated data from EPR spectra of **1**–**4** at 77 K

Compound		Experimental data					Simulated data			
		*g*_⊥_	*A*_⊥_	*g*_‖_	*A*_‖_	*G*	*g*_⊥_	*A*_⊥_	*g*_‖_	*A*_‖_
**1**	Solid state	2.06	–	2.21	–	3.38	2.05	–	2.23	–
**2**	Solid state	2.09	–	–	–	–	2.07	–	2.27	–
**3**	Solid state	2.11	–	–	–	–	2.10	–	2.27	13.10
**4**	Methanol solution	2.05	*	2.27	16.59	4.68	2.05	*	2.27	16.59

*The superhyperfine interaction values that could not be determined due to their complexity

Exchange coupling influences the line shape of the EPR spectra. Values of G > 4.0 indicate negligible interaction, while values < 4.0 correspond to exchange coupling [109]. For **1**, the G value is 3.38, indicating that an exchange interaction occurs, contributing to broadening in the EPR signal. Although **2** shows an isotropic signal, the simulated EPR spectrum indicates an axial symmetry with an estimated value of g_‖_ similar to the experimental value observed for **4** (Table 2, Fig. S5b). Meanwhile, compound **3** presents an EPR spectrum with a low absorption intensity and a different behavior to those observed for **1** and **2** (Fig. S5c). These may be due to a decrease in the spin population due to metal-centered redox processes promoted by dmp, and antiferromagnetic interactions (see magnetic properties section) between the copper complexes, decreasing the magnetic moment between two paramagnetic centers, thus impacting the intensity of the EPR spectra [108].

Finally, as noted in Fig. S5d, the EPR spectrum of **4** shows the typical shape for an axial symmetry with a visible hyperfine interaction in g_‖_, between the unpaired electron and the magnetic nucleus of the Cu(II) ion. Although coupling to the *S* = 1 nitrogen atoms appears to be present in the perpendicular region, determination of the A_*N*_ value was precluded by its complex shape. It is important to note that **4** crystallized in the absence of the [V_4_O_12_]^4−^ system, so it can be assumed that the presence of the tetravanadate affects the total magnetic moment of the paramagnetic centers by decreasing the splitting pattern and signal intensity. This behavior was observed in copper(II) complexes with similar structural features [29].

### Magnetic properties

**Fig. 7:**
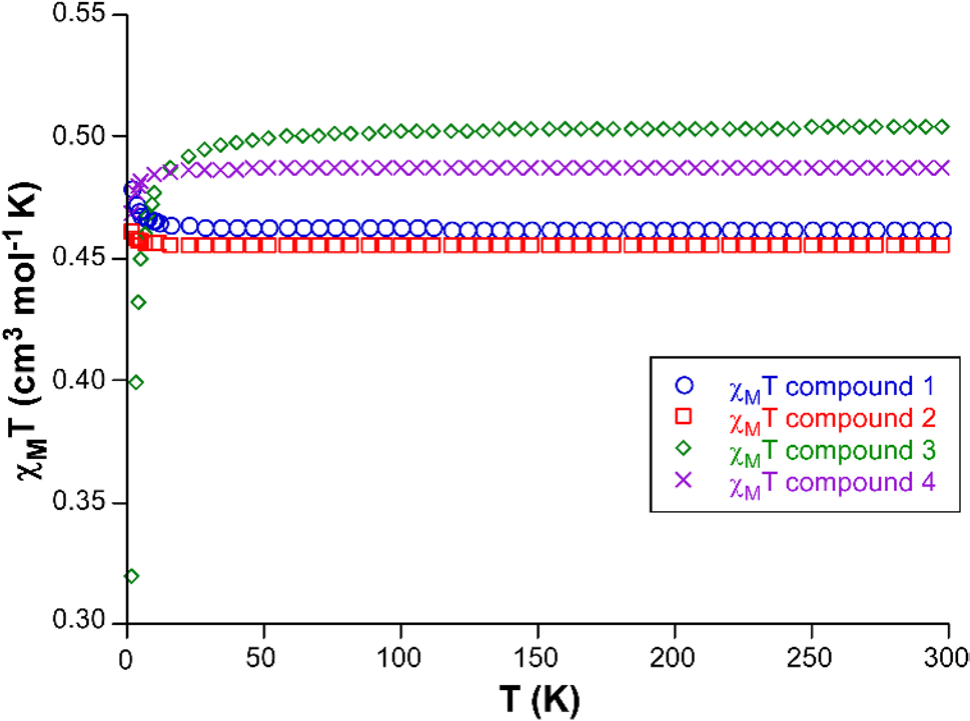
Magnetic susceptibility curves for compounds **1**–**4** in the range of 1.8–300 K

The magnetic properties of these copper(II) complexes were studied by measuring their variable-temperature dc susceptibility (in the 300–1.8 K range) and isothermal magnetization curves of polycrystalline samples. At room temperature, all compounds show higher values of the *χ*_*M*_*T* product (0.461, 0.450, 0.486, and 0.547 cm^3^ mol^−1^ K, respectively, for compounds **1**, **2**, **3**, and **4**) than the value estimated for an uncoupled Cu(II) ion with *g* = 2.01 (0.375 cm^3^ mol^−1^ K), meaning that the latter the gyromagnetic parameters are substantially higher, especially for compound **4**. For comparative purposes, the *χ*_*M*_*T *versus* T* curves are plotted per copper(II) ion, i.e.*,* using their chemical formulae with only one copper(II) atom (Fig. 7). Upon cooling the samples, the *χ*_*M*_*T* product remains almost unchanged as expected for these complexes consisting of isolated entities, with only a slight increase (for **1** and **2**) or decrease (for **3** and **4**), in good agreement with the aforementioned weak interactions expected from their crystal structures.

Starting with compounds **1** and **2**, the increase observed in the *χ*_*M*_*T* product below 30 K up to 1.915 and 0.921 cm^3^ mol^−1^ K for **1** and **2** (considering that compound **1** contains four crystallographically independent Cu(II) centers, whereas **2** contains only two), is indicative of weak but dominant ferromagnetic exchange interactions. Such behavior in these compounds is reinforced by the magnetization curves recorded at low temperatures (Figs. S6, S7), which describe a rapid increase of the magnetization in the 0–3 T range for both compounds, whereas saturation is almost achieved at 6 T and 2 K, reaching values of 4.38 and 2.14 Nμ_B_. Therefore, since compound **2** possesses twice the number of Cu(II) ions per formula unit, the magnetization curves may be considered practically identical.

Similar ferromagnetic behavior observed in both compounds is not surprising considering the presence of carboxylate-bridged {Cu_2_} dimers. All the bridges possess carboxylate groups with anti(equatorial)-syn(axial) coordination modes (with one of the carboxylate oxygen atoms occupying the equatorial plane of the SPY and the other semicoordinated to the apical position), at Cu(II)⋯Cu(II) distances of 5.1–5.2 Å. The unpaired electron is located in the equatorial plane (d_x_^2^_─y_^2^ or d_xy_ orbitals) as corroborated by CASSCF-NEVPT2 calculations performed over all the crystallographically isolated SPY environments of compounds **1** and **2** (vide infra). Under such scheme, the poor overlap between the magnetic orbitals of adjacent Cu(II) ions of the dimers that governs the antiferromagnetic component of the exchange interaction is expected to result in dominant ferromagnetic coupling [110]. Previous compounds containing such Cu‒O‒C‒O‒Cu bridges have shown weak ferromagnetic behavior [111–113].

To estimate the exchange coupling of these compounds, the variable-temperature and magnetization curves were simultaneously fitted through the PHI program [114], considering all the equivalent superexchange pathways existing in each compound (double bridges between Cu1A⋯Cu1B and Cu1C⋯Cu1D in compound **1** and the superexchange Cu1⋯Cu2 bridge in **2**). The best fits were achieved with the set of parameters shown in Table 3 (see Figs. S2, S3). Note that isotropic *g* values could only be fitted to avoid overparametrization. Compound **1** presents more significant anisotropy and ferromagnetic coupling than **2**, which is probably a consequence of the shorter Cu⋯Cu distance and greater overlap of the magnetic orbitals (Cu1A⋯Cu1B in **1** is 5.10 Å and Cu1⋯Cu2 separation in **2** is 5.180 Å).

**Table 3:** Best-fitting and computational results obtained from the analysis of magnetic data of all compounds

Compound	Experimental fitting		Computational data	
	*g*_iso_	*J* (cm^−1^)	CASSCF-NEVPT2 (*g*_iso_)/polyaniso *J* (cm^−1^)	BS computed *J* (cm^−1^)
**1**	2.17(3)	0.11/0.07	2.22/0.36	0.11/0.19
**2**	2.18(2)	0.03	2.23/0.07	0.03
**3**	2.32(5)	− 0.75	2.44/− 0.12	− 0.53
**4**	2.20(3)	− 0.09	2.27	− 0.07

In the case of compounds **3** and **4**, the low-temperature drop of the *χ*_*M*_*T* product suggests their antiferromagnetic character. Such behavior is much more pronounced for **3**, where the product decreases suddenly from 1.0 at 30 K to 0.65 cm^3^ mol^−1^ K at 2 K (considering two Cu(II) ions per formula unit). The M(H) curves also reflect the antiferromagnetic ordering on the more gradual increase of the magnetization according to the increasing field compared to previous compounds. In good agreement, a higher value of the coupling constant is estimated for the combined fitting of *χ*_*M*_*T*(T) and M(H) plots with PHI for **3** relative to **4** (Table 3 and Figs. S8, S9). Moreover, the Cu(II) ions in **3** appear to possess high magnetic anisotropy given the significant value obtained for the *g*_iso_ parameter, which may be derived from the highly distorted coordination environment of the two symmetrically independent ions.

We performed a series of computational calculations using various theoretical approaches to corroborate the previous results further. CASSCF-NEVPT2 calculations were conducted over models of all monomeric complexes grown upon the symmetrically independent Cu(II) ions of the compounds (4 fragments based on Cu1A, Cu1B, Cu1C, and Cu1D in **1**; 2 fragments based on Cu1 and Cu2 for both **2** and **3**; and one fragment for Cu1 in **4**, Figs. S10–S16). Although the value of the *g* parameter is overestimated in all cases, especially for **3**, it describes all the systems adequately. Moreover, two different computational strategies were performed to estimate the value and nature of the coupling constant of the exchange interactions in the structures.

First, the broken-symmetry approach implemented in ORCA, using appropriate models to reproduce the more probable superexchange pathways described for each crystal structure was employed. As summarized in Table 3, the calculated data reproduce fairly well the nature of the exchange interaction and its magnitude in most cases, although the method underestimates the highest interaction found in **3** as it has been previously observed for similar Cu(II) complexes [115]. As observed in the spin-density surfaces for the superexchange pathways of **1** and **2** (Fig. S17a, b), it has the shape of a d_x_^2^_-y_^2^ orbital, in good agreement with the expected location of the unpaired electron density. Moreover, the spin could be transferred through the semicoordinated carboxylate oxygen atoms of the Gly/Lys ligands.

Concerning the antiferromagnetic interaction in **3**, the spin-density extends over the N-containing rings of neighboring dmp ligands that participate in *π*–*π* stacking (Fig. S17c), so that the exchange interaction may proceed through such interaction as supported by other Cu(II)-based complexes [116, 117]. Finally, the weak antiferromagnetic interaction in **4** can also be explained by the long Cu⋯Cl contacts occurring between adjacent complexes in view of the spin-density phase shared by both atoms (Fig. S17d). These exchange interactions have also been studied by the PolyAniso software implemented in ORCA (see results in Table 3 and employed models in Figs. S13–S15). These results confirm the experimentally observed nature of the magnetic interactions among neighboring Cu(II) complexes, although the magnitudes are not as accurate as those calculated by the broken-symmetry approach.

### Stability studies

Copper(II) complexes can be labile and exchange ligands under solution to more thermodynamically stable species [118]. Therefore, the inner coordination sphere observed in the solid state may not be present in the solution, where the complexes exert their biological actions [119]. To analyze the stability of complexes **3** and **4**, we have performed UV–Vis studies over time in the DMEM-F12 culture medium supplemented with 10% fetal bovine serum (FBS) used in the cytotoxicity profiles. The UV–Vis spectra of **3** and **4** show two peaks in the UV region with maximum intensities at 260 and 310 nm, which are assigned to the dmp ligand (Fig. S18) [84, 120]. For both compounds, we observed an absorption band centered at 450 nm, assigned to the metal-binding charge transfer of the reduced species [Cu(dmp)]^+^; this absorption band has been used elsewhere to monitor the formation of dmp-copper systems. This result could indicate the formation of [Cu(dmp)_2_]^+^ from **4** in the culture medium.

To support this hypothesis, we performed electrospray ionization mass spectrometry (ESI/MS) on fresh and aged samples over 2 months at room temperature (Fig. S19). For complex **3**, we identified the fragment corresponding to the homoleptic species [Cu(dmp)_2_]^+^ in both the fresh and aged samples at *m*/*z* = 479.5, indicating that the dmp-copper system is sufficiently stable over time. ESI/MS of compound **4**, both fresh and aged samples, showed the presence of the [Cu(dmp)(Gly)]^+^ fragment at *m*/*z* = 345.1 and *m*/*z* = 345.3, respectively. However, we also observed two peaks in the fresh sample corresponding to the [Cu(dmp)_2_]^+^ at *m*/*z* = 479.1 and [Cu(dmp)_2_Cl]^+^ at *m*/*z* = 514.0, indicating the formation of dmp-Cu(I) adducts and the formation of [Cu(dmp)_2_]^+^ by speciation of **4**. The difference in cytotoxicity of both systems (see below) indicates that at the biological level, both compounds act differently, which can be explained by the presence of tetravanadate in **3**.

### In vitro antiproliferative screening of 3 and 4

The first study to explore the in vitro cytotoxic activity of water-soluble **3** and **4** was performed at 1 µM in a panel of six human cancer lines, i.e., glia (U-251), prostate (PC-3), leukemia (K562), colon (HTC-15), breast (MCF-7), lung (SKLU-1), and a comparative non-cancerous monkey kidney line or COS-7. As shown in Table 4, at this concentration, high cytotoxic activity for both complexes was observed against all cancer lines, with poor selectivity relative to the COS-7 healthy cell line with 100% inhibition. Considering this, a ten-fold reduction in concentration was tested. Tetravanadate-containing **3** presented high cytotoxicity in all cell lines evaluated at this concentration (0.1 µM). In contrast, a decrease in cytotoxicity for **4** against lines U251, PC-3, K562, and HCT-15 was observed, while the MCF-7 line presented an inhibition percentage of 98%. At this concentration, the complex without vanadate inhibited cell proliferation by 53% in the non-cancerous line. Considering the activity of **4** at 0.1 µM concentration, a third assay was performed at a 0.05 µM for both systems.

**Table 4:** % growth inhibitor of compounds **3** and **4** against six human cancer lines at different concentrations

Compound	Concentration (µM)	U251	PC-3	K562	HCT-15	MCF-7	SKLU-1	COS-7
**3**	1	100	100	74.92	100	100	100	100
**4**	1	100	100	86.73	100	100	96.98	100
**3**	0.1	100	100	76.73	100	100	100	79.01
**4**	0.1	76.97	77.49	67.79	72.96	98.16	NA	53.53
**3**	0.05	80.0 ± 3.2	79.2 ± 3.2	95.5 ± 4.3	63.4 ± 3.6	93.1 ± 3.1	73.9 ± 1.8	43.6 ± 3.9
**4**	0.05	63.9 ± 4.3	62.7 ± 4.4	78.4 ± 4.4	53.4 ± 4.8	69.2 ± 4.7	34.7 ± 4.4	35.1 ± 2.2

*NA* non-active

At this concentration, we observed noteworthy results in the cytotoxic profile of both compounds. For **3**, with 43% inhibition of cell growth in the COS-7 line, we observed inhibition of cancer lines K562 and MCF-7 greater than 90%. Moreover, inhibition reached 80% against the malignant glioblastoma tumor (U251, Table 4). For **4**, the best result was inhibition of the K562 line at 78%, while that of the MCF-7 line reached 69%. Concerning the non-cancerous line, this compound exhibited an inhibitory effect of 35%, even lower than **3**. We can argue that compounds **3** and **4** showed cytotoxic activity against MCF-7 and K562, allowing us to perform in vivo biological studies in these cancer cell lines. The cytotoxicity of the compounds was demonstrated in a study carried out on human gingival fibroblasts (HGF) at concentrations of 1 and 2 µM, confirming that the combination with the [V_4_O_12_]^4−^ system presents a higher percentage of inhibition in this primary culture (Table S4). Upon determining the IC_50_ values, it was found that the most active compound was **3** with an IC_50_ value of 12 ± 1.2 nmol against MCF-7, as shown in Table S5.

Vanadium(V) species trigger several physiological effects that depend on their degree of nuclearity, e.g., the simple tetrahedral anion [VO_4_]^3−^ is a known inhibitor of phosphate-dependent enzymes [25, 121, 122]. This activity is related to its role as an antidiabetic agent by inhibiting protein tyrosine phosphatase-1B—an enzyme involved in the positive regulation of some signaling pathways in cancer—allowing tyrosine phosphorylation at insulin receptors IRS-1 and IRS-2, and hence glucose internalization [123–125]. The dimeric [V_2_O_7_]^2−^ species is also recognized to block ATP-dependent K^+^ channels. On the other hand, [V_4_O_12_]^4−^ that must be at a higher concentration at the cellular level than the previous species, has an active role in the formation of reactive oxygen species (ROS) through Fenton-type reactions or by bioreduction of vanadate mediated by glutathione (GSH), which is related to inhibition of growth and cell death [126–128]. These mechanisms of action can presumably work separately or cooperatively, in a manner that is associated to the cytotoxicity of vanadium.

We decided to examine this last hypothesis by evaluating the prooxidant activity of both compounds and, thus, explain the difference in cytotoxicity as a response to the potential role of vanadium species in ROS formation. To this end, a study of the lipid lipoperoxidation induced by compounds **3** and **4** was tested by measuring Thiobarbituric Acid Reactive Substances (TBARS) in rat brain homogenate treated at 1, 10, and 100 µM concentrations. Fig. S20 shows no significant increase in lipid peroxidation was detected in cells treated with compound **3** at 100 µM compared with the control group. At the same time, compound **4** lacking vanadium also showed no significant change in the concentration of TBARS, suggesting that these systems' oxidative activity is not directly related to cytotoxicity. We cannot rule out that a plausible pathway is the inhibition of one of the biological targets mentioned above.

### Wound healing assay

Under homeostatic conditions, cell migration is a physiological property of human cells. However, it becomes a hallmark of cancer under processes linked to invasion and metastasis [129]. Considering the cytotoxic activity of the tested compounds, we decided to investigate their effect on cell migration in three cancer lines, namely, MCF-7, PC-3, and SKLU-1, using a wound-healing assay [130]. In pursuing this goal, a concentration of 5 µM was used for compounds **3** and **4**, considering the difference in the number of cells per well and close to 90% confluence. Thus, a negative control group without any treatment and three positive control groups were established, i.e., two groups under treatment with copper(II) compounds and one group under treatment with mitoxantrone (MTX), a drug used in antineoplastic therapy [131].

**Fig. 8:**
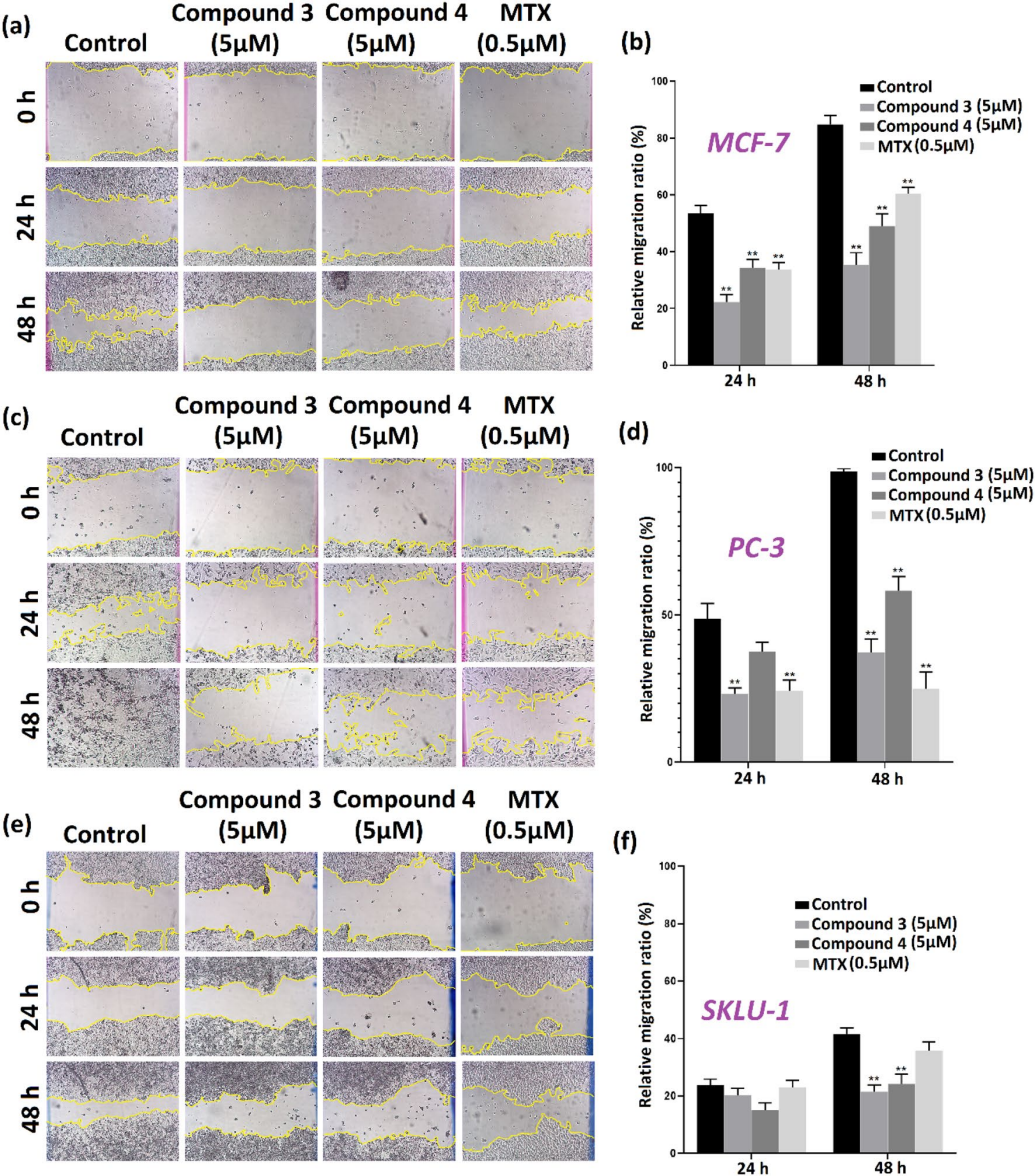
Compounds **3** and **4** inhibit the migration of MCF-7 (**a**, **b**), PC-3 (**c**, **d**), and SKLU-1 (**e**, **f**) cell lines detected by the wound-healing assay. Showed images were captured at 0 min, 24h, and 48h, relative migration ratio (%) was calculated. The data were expressed as mean ± SEM and ** *p* < 0.05 (2way ANOVA)

Figure 8 shows that the untreated control cancer cells migrate rapidly in a time-dependent fashion due to metastatic properties. In contrast, the cells under treatment of **3** and **4** did not migrate at such a high rate, leaving the wound significantly open in almost all cases, indicating that the compounds studied may have an anti-metastatic effect by inhibiting cell migration, as already observed for other copper(II) complexes [132]. However, for SKLU-1, there is no significant difference in any treatment at 24 h concerning the negative control, leading us to suspect that the effect is time-dependent for this cell line (Table S6). Globally, vanadium-containing **3** presented a more significant inhibition in wound closure at 48 h of treatment, presenting a relative migration ratio of 35.2, 37.2, and 21.4% for MCF-7, PC-3, and SKLU-1, respectively, compared to **4**, which showed a relative migration ratio close to 50% for MCF-7 and PC-3 lines, and 24.1% for SKLU-1 (Table S6). The effect of the compounds was comparable in some treatments with the positive control group using MTX.

## Concluding remarks

In this work, we explored the modification of the polyoxovanadate [V_4_O_12_]^4−^ system by introducing copper(II) coordination units to enrich the properties of the compounds and obtain multifunctional materials. Overall, the results presented deserve some general comments.

First, the synthesis strategy was based on the control of pH, one of the most critical parameters to consider within the chemistry of polyoxovanadates. Compounds **1**–**3**, in which tetravanadate is present as ligand or counterion, crystallized at basic pH (around 9), i.e., within the stability region of the [V_4_O_12_]^4−^ system. The structural diversity of **1** and **2** was also influenced by the change of the amino acid and the behavior of these molecules at basic pH. For example, while the glycine acted as a glycinate ion introducing a negative charge to the copper units, the lysine molecule crystallized as a zwitterion; this particularity of the amino acids impacted the structure of these two systems. Complex **3** was obtained as a salt where the [V_4_O_12_]^4−^ anion works as a free species stabilized by two cationic [Cu(dmp)_2_]^2+^ systems. Direct coordination with tetravanadate was not achieved due to the high stability of the tetrahedral [Cu(dmp)_2_]^2+^ units, which prevented, by steric effects, the incorporation of the vanadium oligomer into the coordination sphere. Compound **4** resulted from the variation of pH (from 9 to 6), and although NaVO_3_ was used in the reaction, the [Cu(dmp)(Gly)Cl] species was stabilized. The absence of oligomeric vanadium species in **4** was evident in the IR spectrum, which did not show the typical intense V‒O bands placed in the 1000–450 cm^−1^ range observed for **1**–**3**.

EPR confirmed the paramagnetic nature of all compounds; however, **3** presented a low signal absorption due to the dmp ligand, which has been established as a redox-active ligand platform. Structural analysis of the complexes at the intermolecular level identified short Cu⋯Cu interactions in all cases and Cu⋯O interactions in those compounds with COO^−^ groups acting as bridges between the copper(II) fragments. The magnetic susceptibility values were indicative of weak coupling produced by these interactions. In **1** and **2**, where [V_4_O_12_]^4−^ separates the copper(II) units, magnetic interactions result from bridges mediated by the carboxylate systems from amino acids giving rise to ferromagnetic exchange interactions. In contrast, the significant Cu^…^Cu distances in **3** result in antiferromagnetic behavior where stacking interactions between the aromatic groups of dmp are involved. Although carboxylate groups are present in **4**, they are found to form H-bonds with the –NH groups of neighboring molecules so that the weak antiferromagnetic behavior arises from Cu⋯Cl contacts.

Biological studies were only accessible for **3** and **4** due to their solubility. Cytotoxicity studies showed high activity for both compounds, with the best results at 0.05 µM. **3** showed an inhibition above 90% for the K562 and MCF-7 lines (vs. 43% for the healthy cell line COS-7). For **4**, the best result was achieved in the K562 line with 78% inhibition (35% for the non-cancerous line). Regarding cell migration studies quantified in the wound-healing assay, both compounds inhibit the characteristic proliferative activity of cancer cells. Stability studies of water-soluble **3** and **4** by UV–Vis spectroscopy in cell culture medium confirmed the robustness of **3**, while **4** appears to undergo ligand scrambling over time, resulting partially in the stable species [Cu(dmp)_2_]^+^ that was also identified by electrospray ionization mass spectrometry at *m*/*z* = 479. A critical perspective in this sense would be to study in detail the formation of monomeric or oligomeric vanadium species for the co-crystallized system with the [V_4_O_12_]^4−^ anion, which is known to have various biological effects at the cellular level.

### Supplementary Information

Below is the link to the electronic supplementary material.

Supplementary file 1 (PDF 6070 KB)

## Data Availability

Data will be made available on request.

## References

[1] Zeb Z, Huang Y, Chen L, Zhou W, Liao M, Jiang Y, Li H, Wang L, Wang L, Wang H, Wei T, Zang D, Fan Z, Wei Y (2023). Coord Chem Rev.

[2] Salazar Marcano DE, Savić ND, Abdelhameed SA, De Azambuja F, Parac-Vogt TN (2023) JACS Au 10.1021/jacsau.3c00011 PMC10131212 37124292

[3] Granadeiro CM, Julião D, Ribeiro SO, Cunha-Silva L, Balula SS (2023). Coord Chem Rev.

[4] Budych MJW, Staszak K, Bajek A, Pniewski F, Jastrząb R, Staszak M, Tylkowski B, Wieszczycka K (2023). Coord Chem Rev.

[5] Aureliano M (2022). BioChem.

[6] Zhuang Q, Sun Z, Lin CG, Qi B, Song YF (2023). Inorg Chem Front.

[7] Breibeck J, Gumerova NI, Rompel A (2022). ACS Org Inorg Au.

[8] Anyushin AV, Kondinski A, Parac-Vogt TN (2020). Chem Soc Rev.

[9] Gumerova NI, Rompel A (2018). Nat Rev Chem.

[10] Dolbecq A, Dumas E, Mayer CR, Mialane P (2019). Chem Rev.

[11] Bijelic A, Aureliano M, Rompel A (2019). Angew Chem Int Ed.

[12] Bijelic A, Aureliano M, Rompel A (2018). Chem Commun.

[13] Aureliano M, Gumerova NI, Sciortino G, Garriba E, McLauchlan CC, Rompel A, Crans DC (2022). Coord Chem Rev.

[14] Aureliano M, Gumerova NI, Sciortino G, Garriba E, Rompel A, Crans DC (2021). Coord Chem Rev.

[15] Clemente-Juan JM, Coronado E, Gaita-Ariño A (2012). Chem Soc Rev.

[16] Kortz U, Mueller A, van Slageren J, Schnack J, Dalal NS, Dressel M (2009). Coord Chem Rev.

[17] Li S, Weng Z, Jiang L, Wei R, Su H, Long L, Zheng L, Kong X (2023). Chin Chem Lett.

[18] Long DL, Tsunashima R, Cronin L (2010). Angew Chem Int Ed.

[19] Miras HN, Yan J, Long DL, Cronin L (2012). Chem Soc Rev.

[20] Hasenknopf B (2005). Front Biosci (Landmark Ed).

[21] Liu J, Han Q, Chen L, Zhao J (2016). CrystEngComm.

[22] Oms O, Dolbecq A, Mialane P (2012). Chem Soc Rev.

[23] Gumerova NI, Rompel A (2020). Chem Soc Rev.

[24] Gumerova NI, Rompel A (2023). Sci Adv.

[25] Aureliano M, Crans DC (2009). J Inorg Biochem.

[26] Aureliano M, Ohlin CA, Vieira MO, Marques MPM, Casey WH, De Carvalho LAB (2016). Dalton Trans.

[27] Hayashi Y (2011). Coord Chem Rev.

[28] Sánchez-Lara E, Treviño S, Sánchez-Gaytán BL, Sánchez-Mora E, Castro ME, Melendez-Bustamante FJ, Méndez-Rojas MA, Gonzalez-Vergara E (2018). Front Chem.

[29] Martinez-Valencia B, Corona-Motolinia ND, Sánchez-Lara E, Noriega L, Sánchez-Gaytán BL, Castro ME, Meléndez-Bustamante F, Gonzalez-Vergara E (2020). J Inorg Biochem.

[30] Sánchez-Lara E, Pérez-Benítez A, Treviño S, Mendoza A, Meléndez FJ, Sánchez-Mora E, Bernès S, González-Vergara E (2016). Crystals.

[31] Tabatabaee M, Ahadiat G, Molčanov K (2011). Acta Cryst.

[32] Roman P, San Jose A, Luque A, Gutierrez-Zorrilla JM (1993). Inorg Chem.

[33] Hamilton EE, Fanwick PE, Wilker JJ (2002). J Am Chem Soc.

[34] Wang Q, Yu XL, You WS, Zhao Y, Huang CY, Sun ZG (2007). Inorg Chem Commun.

[35] Paredes-García V, Gaune S, Saldías M, Garland MT, Baggio R, Vega A, Salah El Fallah M, Escuer A, Le Fur E, Venegas-Yazigi D, Spodine E (2008). Inorg Chim Acta.

[36] Thomas J, Agarwal M, Ramanan A, Chernova N, Whittingham MS (2009). Crys Eng Comm.

[37] Martin-Caballero J, San Jose Wery A, Reinoso S, Artetxe B, San Felices L, El Bakkali B, Trautwein G, Alcañiz-Monge J, Vilas JL, Gutiérrez-Zorrilla JM (2016). Inorg Chem.

[38] Sánchez-Lara E, García-García A, González-Vergara E, Cepeda J, Rodríguez-Diéguez A (2021). New J Chem.

[39] Joniaková D, Gyepes R, Rakovský E, Schwendt P, Marek J, Mička Z (2008). Polyhedron.

[40] De Burgomaster P, Zubieta J (2010). Inorg Chimica Acta.

[41] Santini C, Pellei M, Gandin V, Porchia M, Tisato F, Marzano C (2014). Chem Rev.

[42] Marzano C, Pellei M, Tisato F, Santini C (2009). AntiCancer Agents Med Chem.

[43] Tisato F, Marzano C, Porchia M, Pellei M, Santini C (2010). Med Res Rev.

[44] Shobha Devi C, Thulasiram B, Aerva RR, Nagababu P (2018). J Fluores.

[45] Boodram JN, Mcgregor IJ, Bruno PM, Cressey PB, Hemann MT, Suntharalingam K (2016). Angew Chem.

[46] Rivero-Müller A, De Vizcaya-Ruiz A, Plant N, Ruiz L, Dobrota M (2007). Chem Biol Interact.

[47] Leal-García M, García-Ortuño L, Ruiz-Azuara L, Gracia-Mora I, Luna-del Villar J, Sumano H (2007). Basic Clin Pharmacol Toxicol.

[48] Wehbe M, Leung AW, Abrams MJ, Orvig C, Bally MB (2017). Dalton Trans.

[49] Bain GA, Berry JF (2008). J Chem Educ.

[50] Li J, Wei C, Guo D, Wang C, Han Y, He G, Zhang J, Huang X, Hu C (2020). Dalton Trans.

[51] Larrea ES, Mesa JL, Pizarro JL, Arriortua MI, Rojo T (2007). J Solid State Chem.

[52] CrysAlisPro, version 1.171.36.32; Oxford Diffraction Ltd., Abingdon, UK, 2013

[53] Clark RC, Reid JS (1995). Acta Cryst.

[54] Sheldrick GM (2015). Acta Cryst.

[55] Sheldrick GM (2015). Acta Cryst.

[56] Farrugia LJ (2012). J Appl Cryst.

[57] Macrae CF, Edgington PR, McCabe P, Pidcock E, Shields GP, Taylor R, Towler M, van der Streek J (2006). Appl Crystallogr.

[58] Neese F (2022). Wiley Interdiscip Rev Comput Mol Sci.

[59] Neese F, Wennmohs F, Becker U, Riplinger C (2020). J Chem Phys.

[60] Neese F (2012). Wiley Interdiscip Rev Comput Mol Sci.

[61] Beeke AD (1993). J Chem Phys.

[62] van Wüllen C (1998). J Chem Phys.

[63] Weigend F, Ahlrichs R (2005). Phys Chem Chem Phys.

[64] Weigend F (2006). Phys Chem Chem Phys.

[65] Hellweg A, Hättig C, Höfener S, Klopper W (2007). Theor Chem Acc.

[66] Angeli C, Borini S, Cestari M, Cimiraglia R (2004). J Chem Phys.

[67] Ungur L, Chibotaru LF (2017). Chem Eur J.

[68] Chibotaru LF, Ungur L (2012). J Chem Phys.

[69] Domínguez M, Nieto A, Marín JC, Keck AS, Jeffery E, Céspedes CL (2005). J Agric Food Chem.

[70] Lowry OH, Rosebrough NJ, Farr AL, Randall RJ (1951). J Biol Chem.

[71] Floyd RA (1999). Antioxidants, oxidative stress, and degenerative neurological disorders. Proc Soc Exp Biol Med.

[72] Ohkawa H, Ohishi N, Yagi K (1979). Anal Biochem.

[73] Dotan Y, Lichtenberg D, Pinchuk I (2004). Prog Lipid Res.

[74] Fernández J, Pérez-Álvarez JA, Fernández-López JA (1997). Food Chem.

[75] Esterbauer H, Cheeseman KH (1990). Methods Enzymol.

[76] Monakhov KY, Bensch W, Kögerler P (2015). Chem Soc Rev.

[77] Martinez-Valencia B, Corona-Motolinia ND, Sánchez-Lara, Sánchez-Gaytán B, Cerro-López M, Mendoza A, Castro Sánchez ME, Melendez-Bustamante F, Gonzalez-Vergara E (2020) Crystals 10:492

[78] Alikhani M, Hakimi M, Moeini K, Mashreghi M, Eigner V, Dusek M (2020). J Mol Struc.

[79] Bencini A, Lippolis V (2010). Coord Chem Rev.

[80] Constable EC, Housecroft CE (2019). Molecules.

[81] D'Andrea G, Di Nicolantonio J (2002). Chem Ed.

[82] Nolting D, Aziz EF, Ottosson N, Faubel M, Hertel IV, Winter B (2007). J Am Chem Soc.

[83] Leandri V, Daniel Q, Chen H, Sun L, Gardner JM, Kloo L (2018). Inorg Chem.

[84] Mitrofin S, Chan EJ, Healy PC, Marinelli A, Ngoune J, Perrinari CI, Pettinari R, Somers N, Skelton BW, White AH (2008). Inorg Chim Acta.

[85] Pinsky M, Avnir D (1998). Inorg Chem.

[86] Addison AW, Rao TN, Reedijk J, van Rijn J, Vershoor GC (1984). J Chem Soc Dalton trans.

[87] Vitoria P, San José Wery A, San Felices L, Bravo-García L, Ruiz-Bilbao E, Laza JM, Vilas JL, Gutierrez-Zorrila JM (2022). Materials.

[88] MacRae CF, Sovago I, Cottrell SJ, Galek PTA, McCabe P, Pidcock E, Platings M, Shields GP, Stevens JS, Towler M, Wood PA (2020). J Appl Crystallogr.

[89] Bruno IJ, Cole JC, Kessler M, Luo J, Motherwell WDS, Purkis LH, Smith BR, Taylor R, Cooper RI, Harris SE, Orpen AG (2004). J Chem Inf Comput.

[90] Yang L, Powell DR, Houser RP (2007). Dalton Trans.

[91] Itoh S, Kishikawa N, Suzuki T, Takagi HD (2005). Dalton Trans.

[92] Flörke U, Stührenberg K, Bauer M (2018) CCDC 1862170: experimental crystal structure determination. 10.5517/ccdc.csd.cc20hr09

[93] Lin X, Hou C, Li H, Weng Z (2016). Chem Eur J.

[94] Watton SP (2009). Acta Crystallogr Sect E Struct Rep Online.

[95] Tran D, Skelton BW, White AH, Laverman LE, Ford PC (1998). Inorg Chem.

[96] Smith CS, Mann KR (2009). Chem Mater.

[97] Kovalevsky AY, Gembicky M, Novozhilova IV, Coppens P (2003). Inorg Chem.

[98] Zheng S, Gembicky M, Messerschmidt M, Dominiak PM, Coppens P (2006). Inorg Chem.

[99] Tye JW, Weng Z, Giri R, Hartwig HF (2010). Angew Chem Int Ed.

[100] Etter MC (1990). Acc Chem Res.

[101] Nakamoto K (2009). Infrared and Raman spectra of inorganic and coordination compounds, part B: applications in coordination organometallic, and bioinorganic chemistry.

[102] Corona-Motolinia ND, Martínez-Valencia B, Noriega L, Sánchez-Gaytán BL, Mendoza A, Meléndez-Bustamante FJ, Castro ME, González-Vergara E (2021). Metals.

[103] Kucková L, Jomová K, Švorcová A, Valko M, Segľa P, Moncoľ J, Kožíšek J (2015). Molecules.

[104] Muslim M, Ahmad M, Arish M, Alam MJ, Alarifi A, Afzal M, Sepay N, Ahmad S (2022). J Mol Struct.

[105] Garribba E, Micera G (2006). J Chem Edu.

[106] Subramanian PS, Suresh E, Dastidar P, Waghmode S, Srinivas D (2001). Inorg Chem.

[107] Pilbrow JR, Toy AD, Smith TD (1969). J Chem Soc A.

[108] Brustolon M, Giamello E (2009). Electron paramagnetic resonance.

[109] Procter IM, Hathaway BJ, Nicholls P (1968). J Chem Soc A.

[110] Kahn O (1993). Molecular magnetism.

[111] Delgado FS, Sanchiz J, Ruiz-Pérez C, Lloret F, Julve M (2003). Inorg Chem.

[112] Perez-Yanez S, Castillo O, Cepeda J, García-Terán JP, Luque A, Román P (2009). Eur J Inorg Chem.

[113] Podjed N, Modec B, Clérac R, Rouzières M, Alcaide MM, López-Serrano J (2022). New J Chem.

[114] Chilton NF, Anderson RP, Turner LD, Soncini A, Murray KS (2013). J Comput Chem.

[115] Yadav A, Bieńko A, Bieńko DC, Wojtala D, Siddiqui KA (2022). Polyhedron.

[116] Li H, Zhang SG, Xie LM, Yu L, Shi JM (2011). J Coord Chem.

[117] Havlíček L, Herchel R, Nemec I, Neugebauer P (2022). Polyhedron.

[118] Krasnovskaya O, Naumov A, Guk D, Gorelkin P, Erofeev A, Beloglazkina E, Majouga A (2020). Int J Mol Sci.

[119] Alvarez N, Viña D, Leite CM, Mendes LF, Batista AA, Ellena J, Costa-Filho AJ, Facchin G (2020). J Inorg Biochem.

[120] Itoh S, Kishikawa N, Suzuki T, Takagi HD (2005). Dalton Trans.

[121] Treviño S, Díaz A, Sánchez-Lara E, Sanchez-Gaytan BL, Perez-Aguilar JM, González-Vergara E (2019). Biol Trace Elem Res.

[122] Crans DC, Smee JJ, Gaidamauskas E, Yang L (2004). Chem Rev.

[123] Huyer G, Liu S, Kelly J, Moffat J, Payette P, Kennedy B, Tsaprailis G, Gresser MJ, Ramachandran C (1997). J Biol Chem.

[124] Johnson TO, Ermolieff J, Jirousek MR (2002). Nat Rev Drug Discov.

[125] Östman A, Hellberg C, Böhmer FD (2006). Nat Rev Cancer.

[126] Silva-Nolasco AM, Camacho L, Saavedra-Díaz RO, Hernández-Abreu O, León IE, Sánchez-Lombardo I (2020). Inorganics.

[127] Hosseini MJ, Shaki F, Ghazi-Khansari M, Pourahmad J (2013). Metallomics.

[128] Evangelou AM (2002). Crit Rev Oncol Hematol.

[129] Friedl P, Wolf K (2003). Nat Rev Cancer.

[130] Rodriguez LG, Wu X, Guan JL, Guan JL (2005). Wound-Healing Assay. Cell migration. Methods in molecular biology™.

[131] Evison BJ, Sleebs BE, Watson KG, Phillips DR, Cutts SM (2016). Med Res Rev.

[132] Nagababu P, Barui AK, Thulasiram B, Devi CS, Satyanarayana S, Patra CR, Sreedhar B (2015). J Med Chem.

