# Supplementary material for: Effects of the tetravanadate [V4O12]4− anion on the structural, magnetic, and biological properties of copper/phenanthroline complexes
Source: J Biol Inorg Chem. 2024 Jan 4;29(1):139–58. doi: 10.1007/s00775-023-02035-9 (PMC11001746; doi:10.1007/s00775-023-02035-9)
Supplement: Supplementary file 1 — (PDF 6070 KB) [file 775_2023_2035_MOESM1_ESM.pdf]

Supplementary Material

Effects of the tetravanadate $[V_4O_{12}]^{4-}$ anion on the structural, magnetic, and biological properties of copper/phenanthroline complexes

Eduardo Sánchez-Lara,[1*] Roberto Favela,[1] Kitze Tzian,[1] Brian Monroy-Torres,[1] Adriana Romo-Pérez,[1] María Teresa Ramírez-Apan[1], Marcos Flores-Alamo[2], Antonio Rodríguez-Diéguez[3], Javier Cepeda[4] and Ivan Castillo[1*]

[1]Insituto de Química, Universidad Nacional Autónoma de México, Circuito Interior, CU, Ciudad de México, 04510, Mexico.

[2]Facultad de Química, Universidad Nacional Autónoma de México, Circuito Exterior, CU, Ciudad de México, 04510 Mexico.

[3]Departamento de Química Inorgánica, Facultad de Ciencias, Universidad de Granada, Avda. Fuentenueva, 18071 Granada, Spain.

[4]Departamento de Química Aplicada, Facultad de Química, Universidad del País Vasco UPV/EHU, 10018 Donostia-San Sebastian, Spain.

*Correspodence: esl_24@hotmail.com; joseivan@unam.mx

Table S1. CShMs for the coordination environment of the Cu(II) centres of **1** and **2**. The lowest SHAPE values for each ion are shown highlighted in grey.

Codes:

PP-5 1 D5h Pentagon

vOC-5 2 C4v Vacant octahedron

TBPY-5 3 D3h Trigonal bipyramid

SPY-5 4 C4v Spherical square pyramid

JTBPY-5 5 D3h Johnson trigonal bipyramid J12

Structure [ML5]	PP-5	vOC-5	TBPY-5	SPY-5	JTBPY-5
Compound **1**					
**Cu1A**	30.554	1.240	5.301	0.984	7.968
**Cu1B**	30.069	1.012	6.012	0.981	8.504
**Cu1C**	29.716	1.121	6.202	1.193	8.837
**Cu1D**	31.034	1.201	5.225	0.889	7.958
Compound **2**					
**Cu1**	31.760	1.256	5.606	0.892	8.307
**Cu2**	28.504	2.518	2.864	1.447	5.859
Compound **4**					
**Cu1**	26.180	2.088	6.347	1.904	9.328

Table S2. Selected bond distances (Å) and angles (deg) of compounds 1-4.

**Compound 1**		**Compound 2**		**Compound 3**		**Compound 4**	
Cu1A—N1A	1.970(5)	Cu1—N1	1.958(9)	Cu1—N1	2.042(3)	Cu1-N1	2.022(2)
Cu1A—N2A	1.999(5)	Cu1—N2	2.001(8)	Cu1—N2	2.038(3)	Cu1-N2	2.271(2)
Cu1A—N3A	1.993(5)	Cu1—N3	1.986(10)	Cu1—N3	2.032(3)	Cu1-N3	2.014(2)
Cu1A—O1A	1.967(4)	Cu1—O13	1.926(15)	Cu1—N4	2.050(3)	Cu1-O1	1.9530(19)
Cu1A—O2	2.267(4)	Cu1—O12	2.306(8)	Cu2—N5	2.062(3)	Cu1-Cl1	2.3097(7)
Cu1B—N1B	1.984(5)	Cu2—N5	1.972(8)	Cu2—N6	2.047(3)		
Cu1B—N2B	1.970(5)	Cu2—N6	2.001(8)	Cu2—N7	2.062(3)		
Cu1B—N3B	1.989(4)	Cu2—N7	2.007(9)	Cu2—N8	2.051(3)		
Cu1B—O1B	1.982(4)	Cu2—O17	1.947(7)				
Cu1B—O8	2.269(4)	Cu2—O6	2.223(13)				
Cu1C—N1C	2.010(5)						
Cu1C—N2C	1.968(5)						
Cu1C—N3C	1.977(5)						
Cu1C—O1C	1.981(4)						
Cu1C—O1W	2.311(5)						
Cu1D—N1D	2.010(5)						
Cu1D—N2D	1.984(5)						
Cu1D—N3D	1.992(5)						
Cu1D—O1D	1.960(4)						
Cu1D—O2W	2.281(5)						
V1—O1	1.784(4)	V1—O3	1.798(8)	V1—O3	1.805(2)	O1-Cu1-N1	171.24(9)
V1—O2	1.646(4)	V1—O4	1.776(8)	V1—O4	1.805(2)	O1-Cu1-N2	110.06(8)
V1—O4	1.786(4)	V2—O3	1.790(7)	V2—O3	1.788(2)	N3-Cu1-N2	98.64(9)
V2—O4	1.790(4)	V2—O7	1.871(13)	V2—O4	1.772(2)	N1-Cu1-N2	78.64(8)
V2—O7	1.791(4)	V2—O6	1.655(14)			N1-Cu1-Cl1	90.41(6)
V3—O7	1.799(4)	V3—O7	1.550(15)				
V3—O8	1.654(4)	V3—O10	1.794(13)				
V3—O10	1.795(4)	V4—O10	1.786(12)				
V4—O1	1.802(4)	V4—O4	1.841(7)				
V4—O10	1.798(4)	V4—O12	1.594(8)				
V1—O1-V4	139.1(2)	V1—O4-V4	138.8(4)				
V1—O4-V2	159.3(3)	V1—O3—V2	146.8(4)	V1—O3—V2	138.43(14)		
V2—O7-V3	143.2(2)	V2—O7—O3	153.0(8)	V2—O4—V1	144.16(15)		
V3—O10-V4	149.4(3)	V3—O10—V4	151.6(8)				

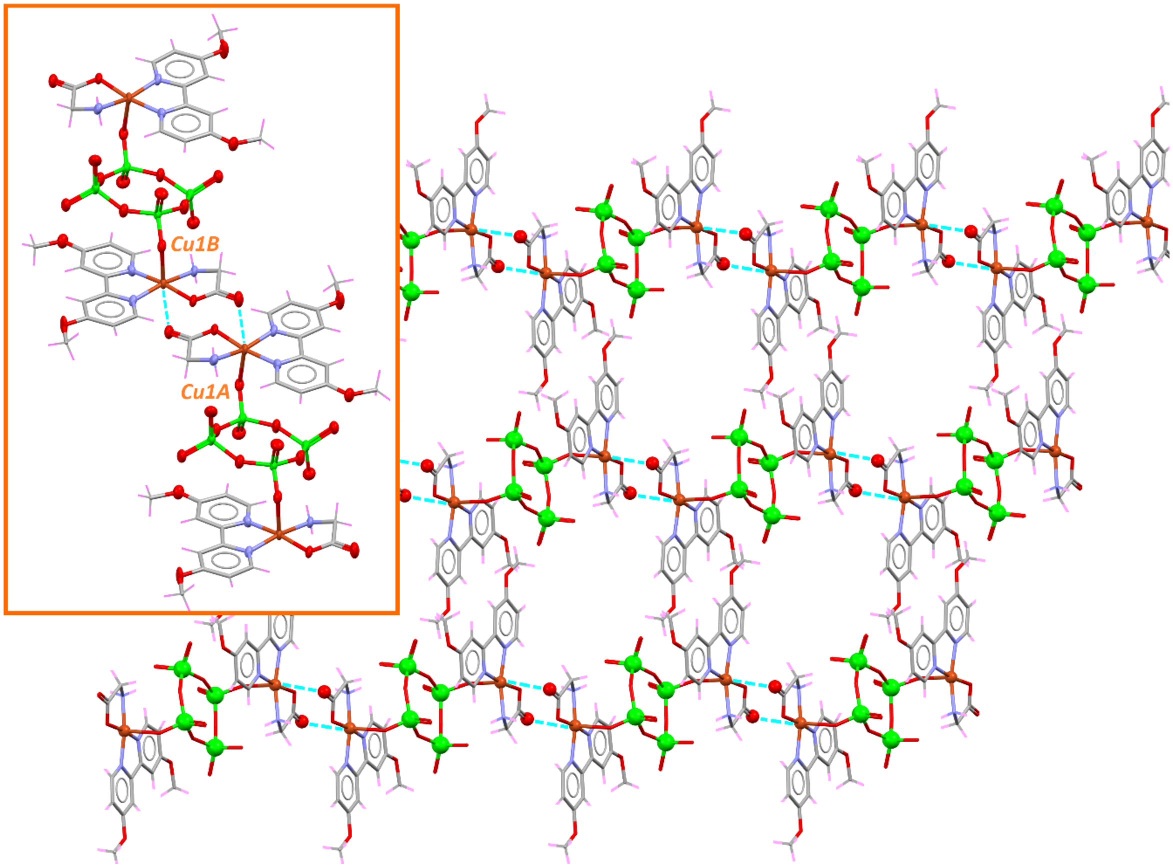

Figure S1. Crystal packing of **1** showing the stacking between the $[Cu(dmbp)(Gly)]_2[V_4O_{12}]$ units. The inset highlights the intermolecular interactions between the Cu^{2+} ions and the COO^- groups.

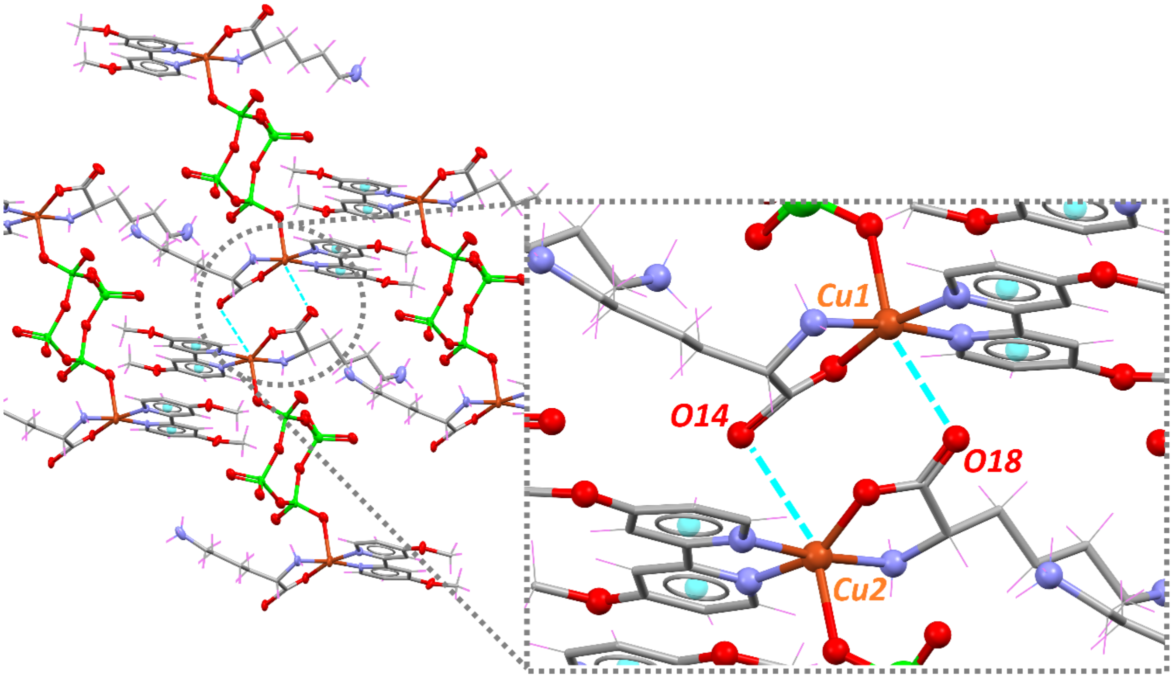

Figure S2. Crystal packing of **2**. The expanded view highlights the intermolecular interactions between two independent copper(II) centers.

Table S3. CShMs for the coordination environment of the Cu(II) centres of **3**. The lowest SHAPE values for each ion are shown highlighted in grey.

Codes:

SP-4 1 D4h Square

T-4 2 Td Tetrahedron

SS-4 3 C2v Seesaw

vTBPY-4 4 C3v Vacant trigonal bipyramid

**Structure [ML4]**	**SP-4**	**T-4**	**SS-4**	**vTBPY-4**
**Cu1**	17.621	6.480	6.569	9.190
**Cu2**	18.890	6.678	7.495	9.183

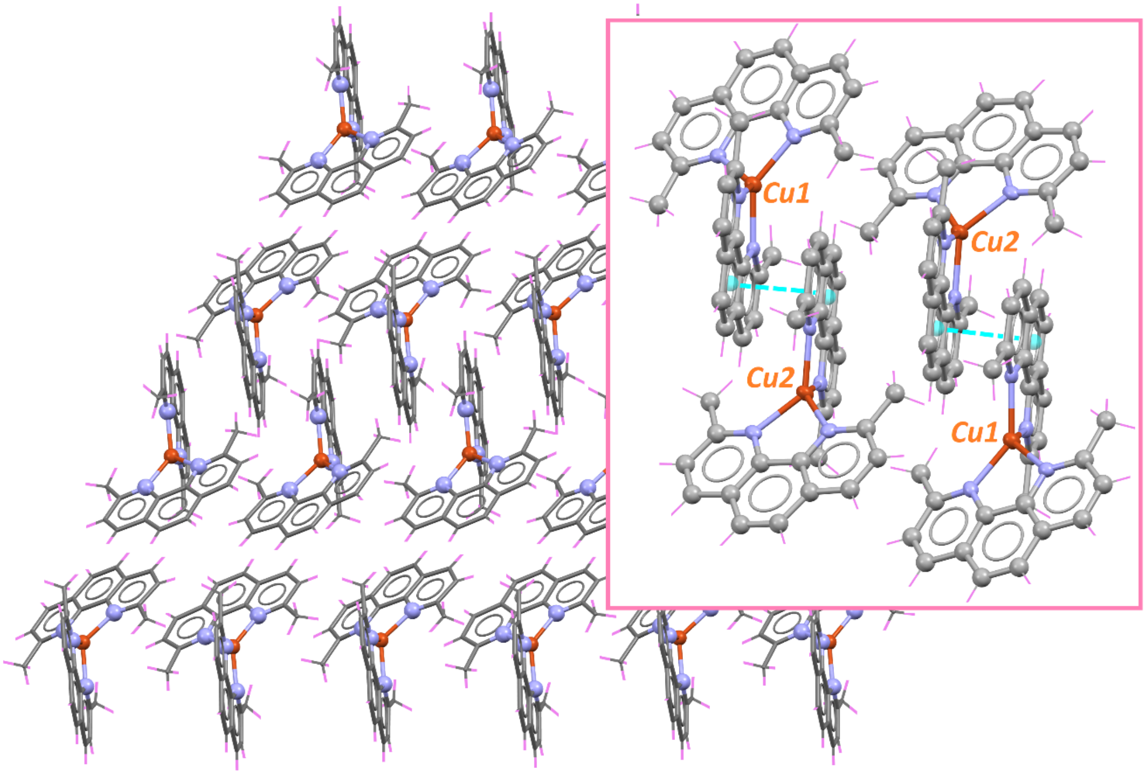

Figure S3. Crystal packing of **3** showing the arrangement of the $[Cu(dmp)]^{2+}$ dications. The inset displays the π-π stacking interactions between dmp ligands.

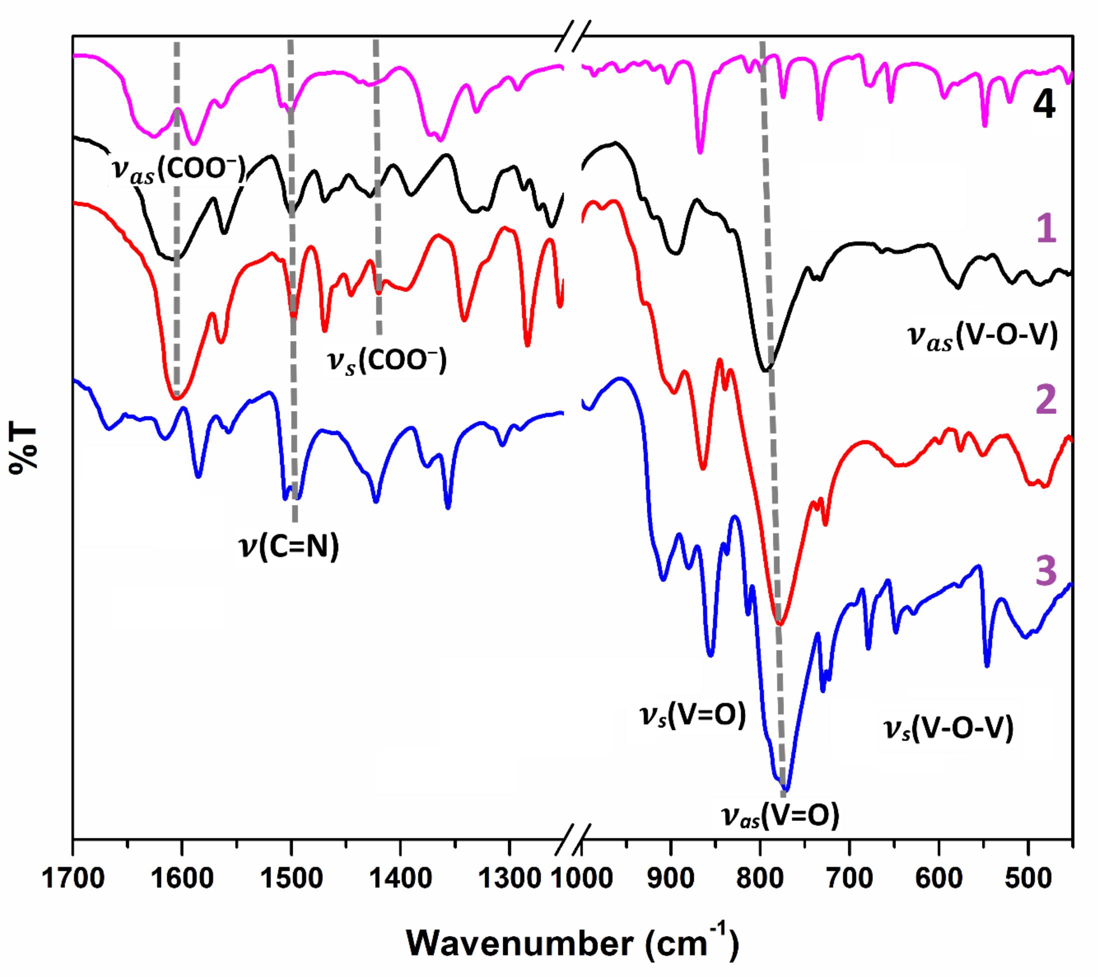

Figure S4. ATR-FTIR spectra of **1**-**4** in the range of 1700-450 cm^{-1}.

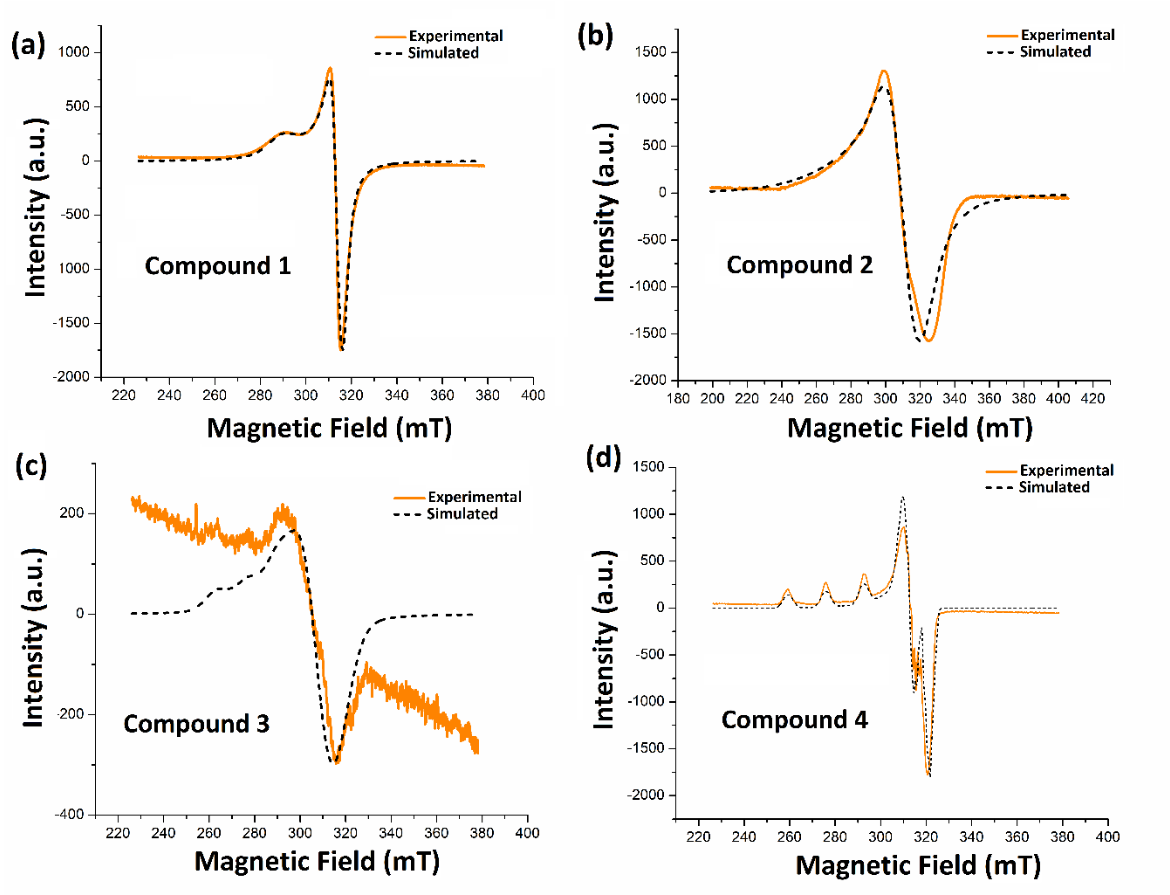

Figure S5. Experimental and simulated EPR spectra of **1**-**4** at 77 K. Compounds **1**-**3** were measured in the solid state, while **4** was measured in a methanol solution.

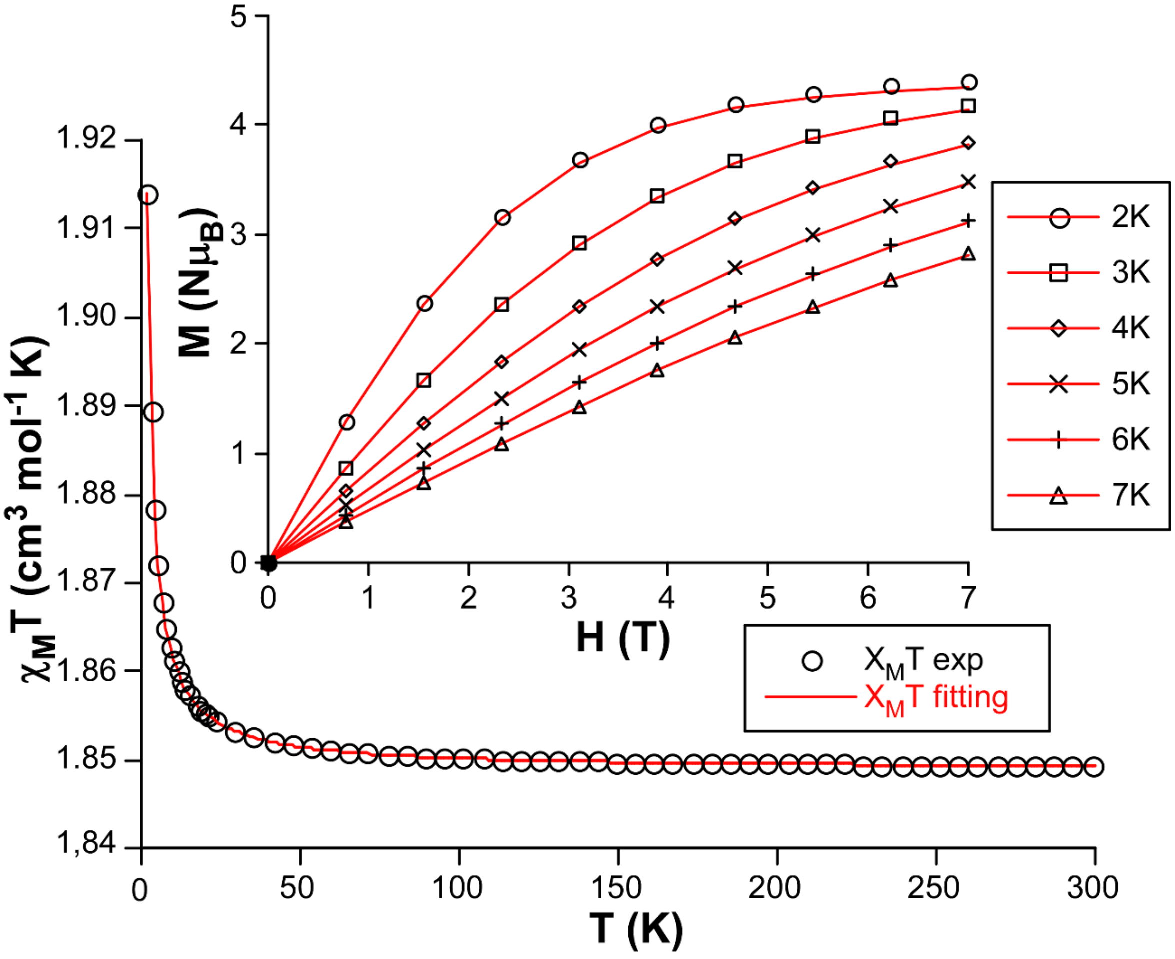

Figure S6. Best fitting of $\chi_M T$(T) and M(H) plots of **1**.

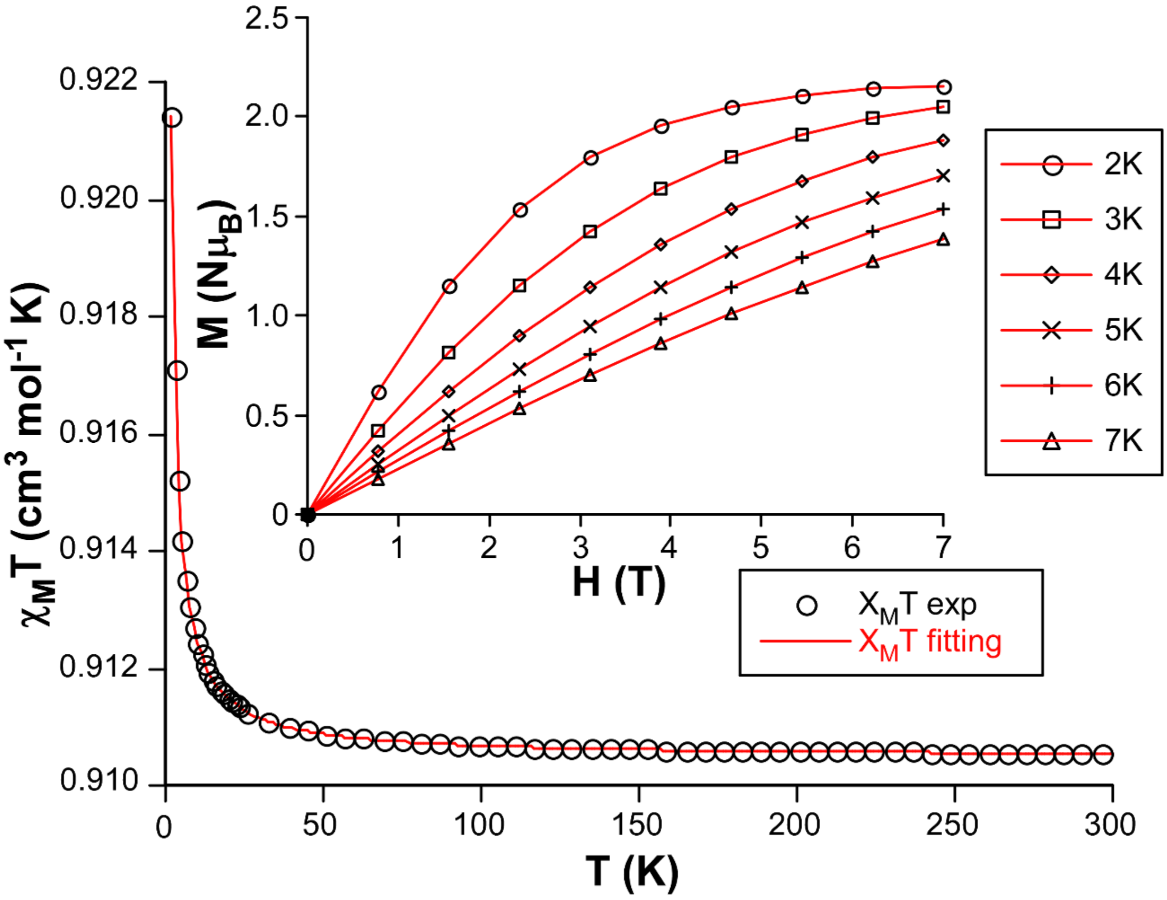

Figure S7. Best fitting of $\chi_M T$(T) and M(H) plots of **2**.

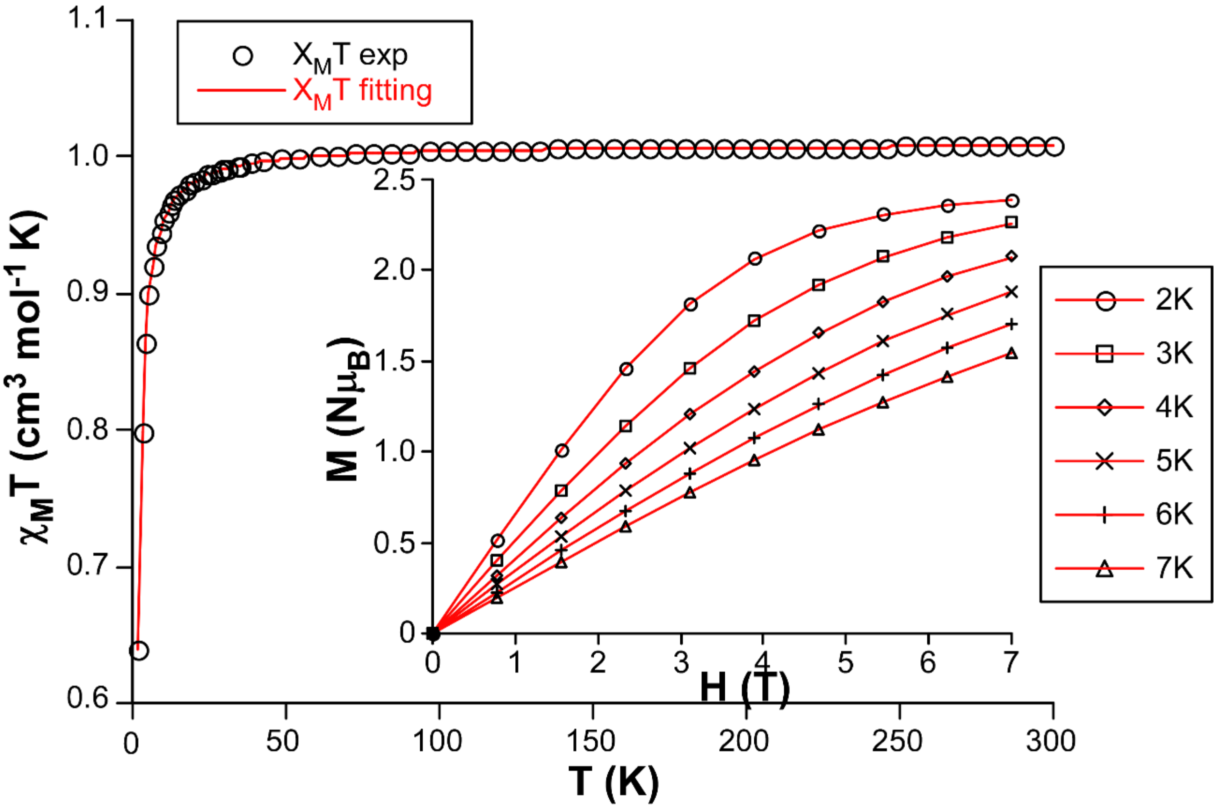

Figure S8. Best fitting of $\chi_M T$(T) and M(H) plots of **3**.

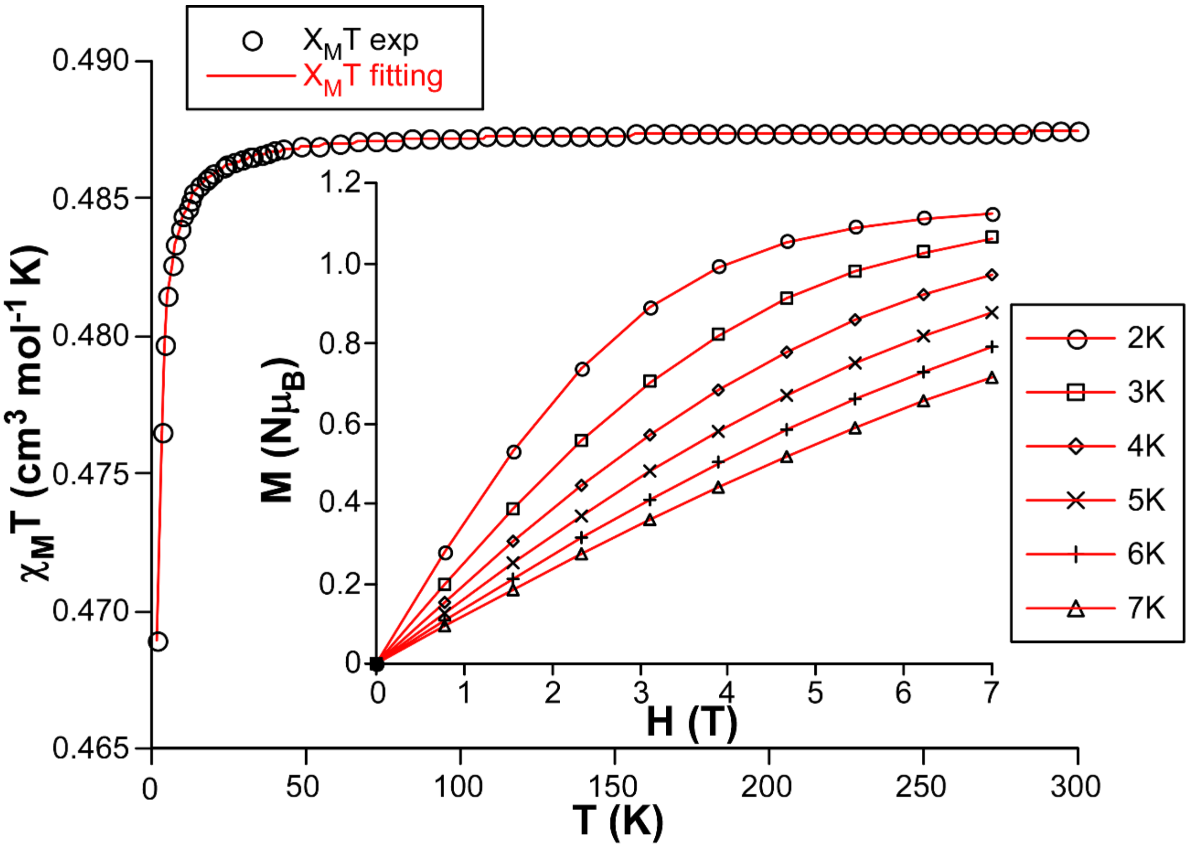

Figure S9. Best fitting of $\chi_M T$(T) and M(H) plots of **4**.

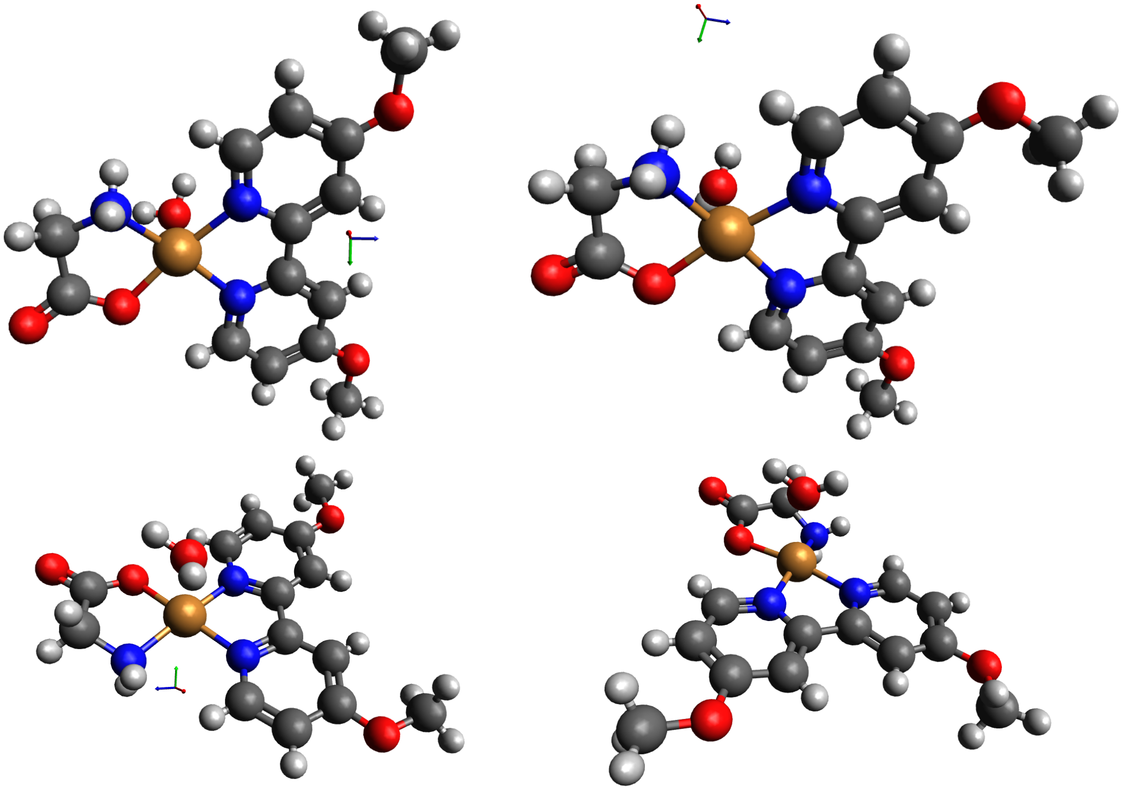

Figure S10. Models of monomeric complexes used in **1** to estimate the magnetic properties by means of CASSCF-NEVPT2-Single_Aniso calculations.

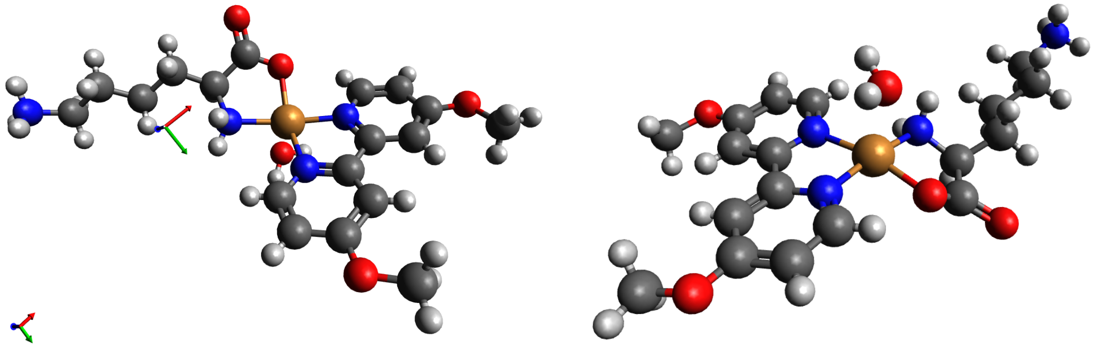

Figure S11. Models of monomeric complexes used in **2** to estimate the magnetic properties by means of CASSCF-NEVPT2-Single_Aniso calculations.

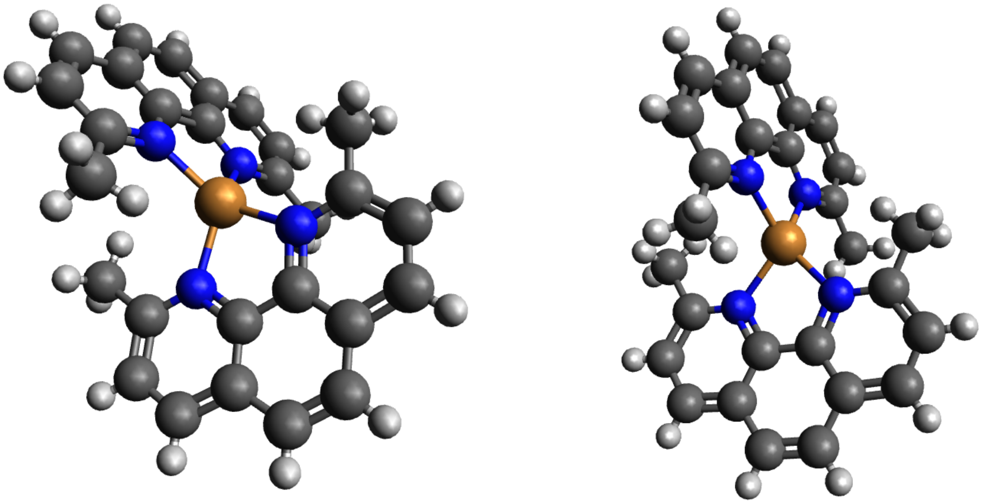

Figure S12. Models of monomeric complexes used in **3** to estimate the magnetic properties by means of CASSCF-NEVPT2-Single_Aniso calculations.

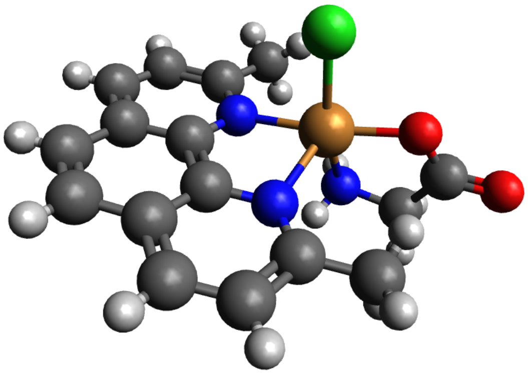

Figure S13. Models of monomeric complexes used in **4** to estimate the magnetic properties by means of CASSCF-NEVPT2-Single_Aniso calculations.

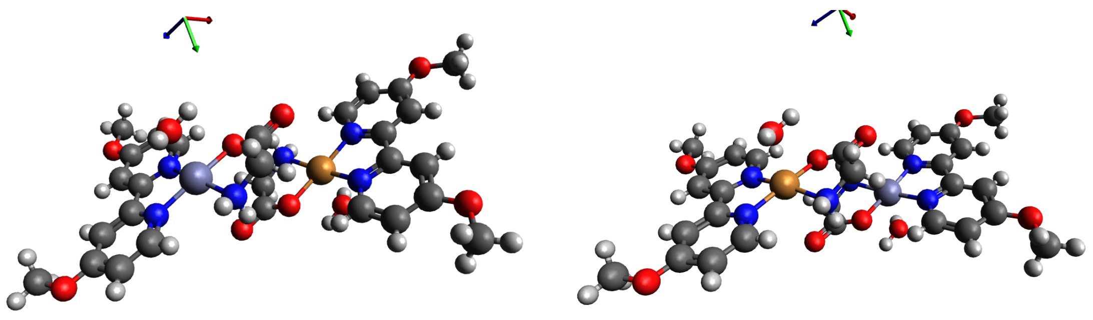

Figure S14. View of the dimeric model used in **1** to estimate the magnetic coupling constant by means of Poly_Aniso calculation on top of CASSCF-NEVPT2-Single_Aniso calculations performed on the individual Cu(II) complexes. Two equivalent models have been employed using Zn(II) (grey) and Cu(II) (golden yellow) (at the left) and Cu(II) and Zn(II) (at the right) as metal centers in the monomers.

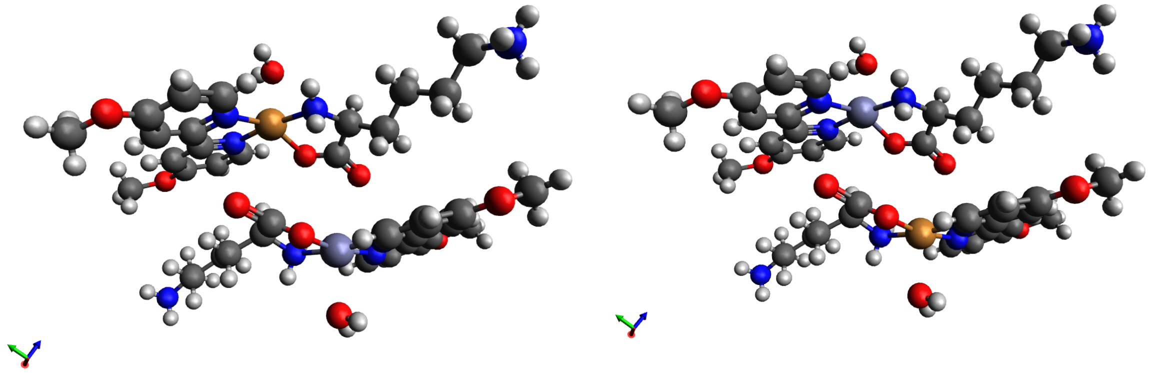

Figure S15. View of the dimeric model used in **2** to estimate the magnetic coupling constant by means of Poly_Aniso calculation on top of CASSCF-NEVPT2-Single_Aniso calculations performed on the individual Cu(II) complexes. The same color codes are employed for Zn(II) and Cu(II) atoms.

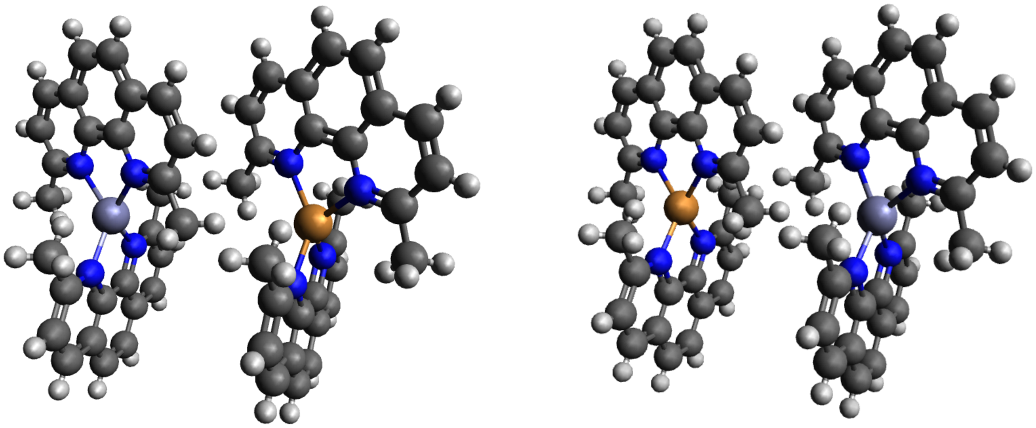

Figure S16. View of the dimeric model used in **3** to estimate the magnetic coupling constant by means of Poly_Aniso calculation on top of CASSCF-NEVPT2-Single_Aniso calculations performed on the individual Cu(II) complexes. The same color codes are employed for Zn(II) and Cu(II) atoms.

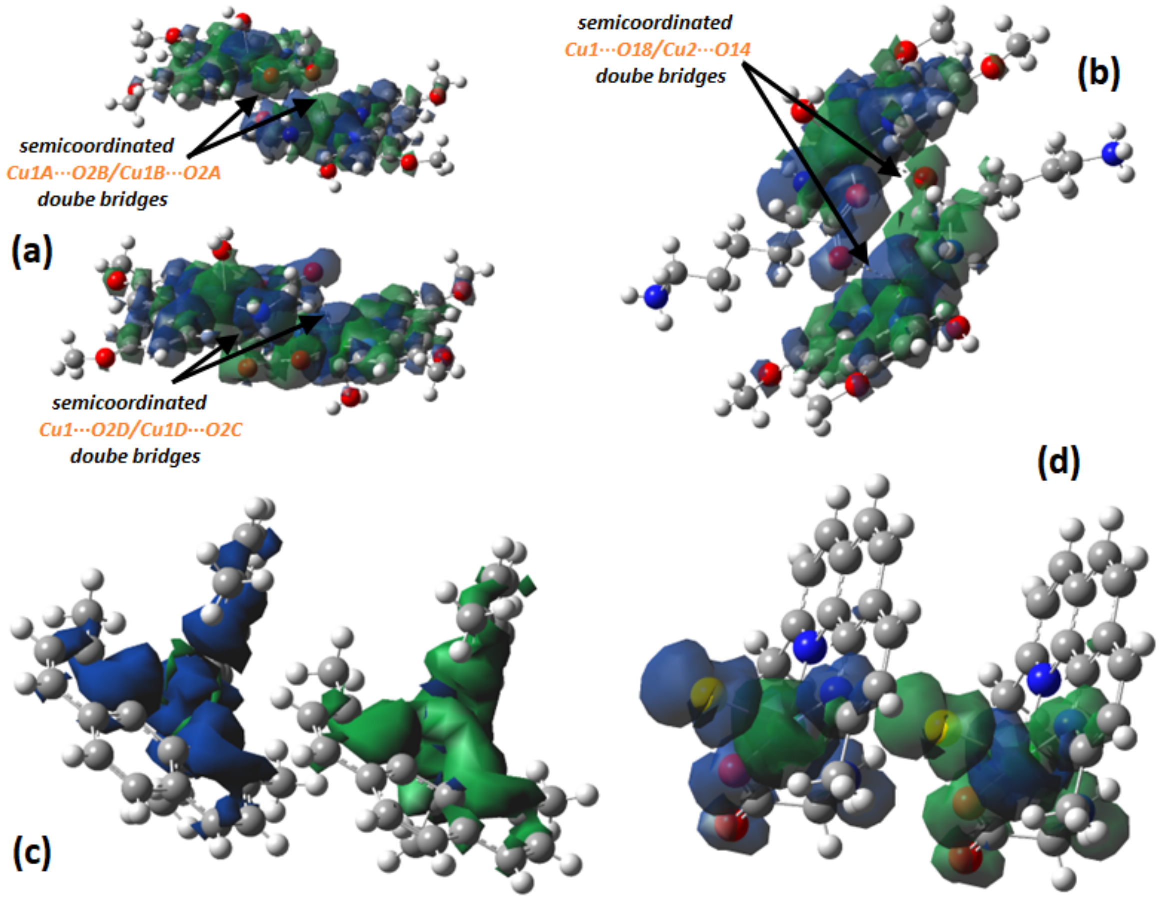

Figure S17. (a) Spin-density surfaces shown for the main superexchange pathways found in **1**: Cu1A···Cu1B (top) and Cu1C···Cu1D (bottom). (b) The superexchange pathway (Cu1···Cu2) found in **2**; the dashed lines represent the semicoordinated atoms that bring about {Cu_2} entities in both structures. (c) Spin-density surfaces drawn upon a dimeric complex based on two isolated Cu(II) entities to represent the superexchange pathway (Cu1···Cu2) found in **3**. (c) the superexchange pathway (Cu1···Cu2) found in **4**.

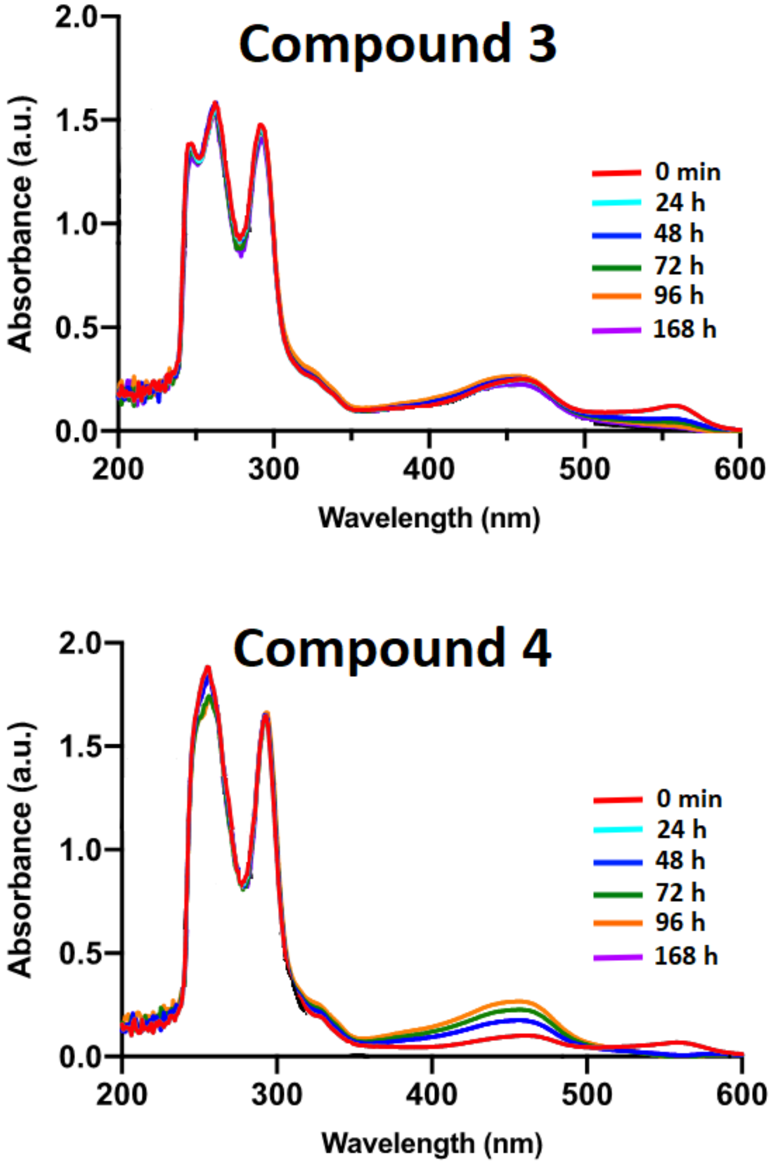

Figure S18. Time dependent UV–Vis spectra of **3** and **4**, in cell culture media.

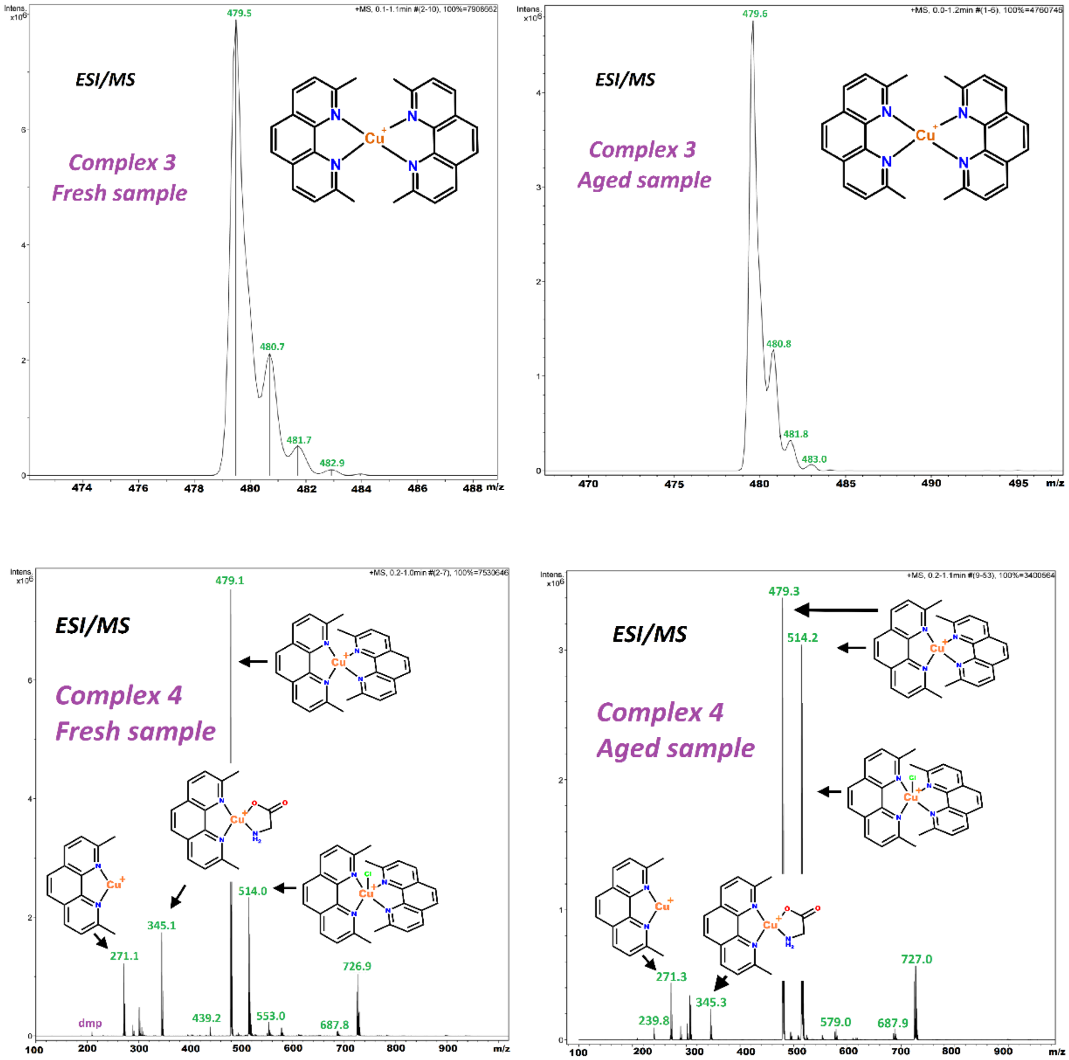

Figure S19. Comparison of compounds **3** and **4** ESI mass spectra as fresh and aged samples.

Table S4. % growth inhibitor of **3** and **4** against primary fibroblast culture

Compound	1 µM	2µM
**3**	78.73±3.6	93.98±3.3
**4**	54.97±3.1	76.35±7.6

Table S5. IC_{50} values (nmol) of compounds **3** and **4** towards PC-3. MCF-7, and SKLU-1.

Compounds	PC-3	MCF-7	SKLU-1
**3**	25±2.0	12±1.2	29±1.0
**4**	20±2.0	28±0.4	37±3.0

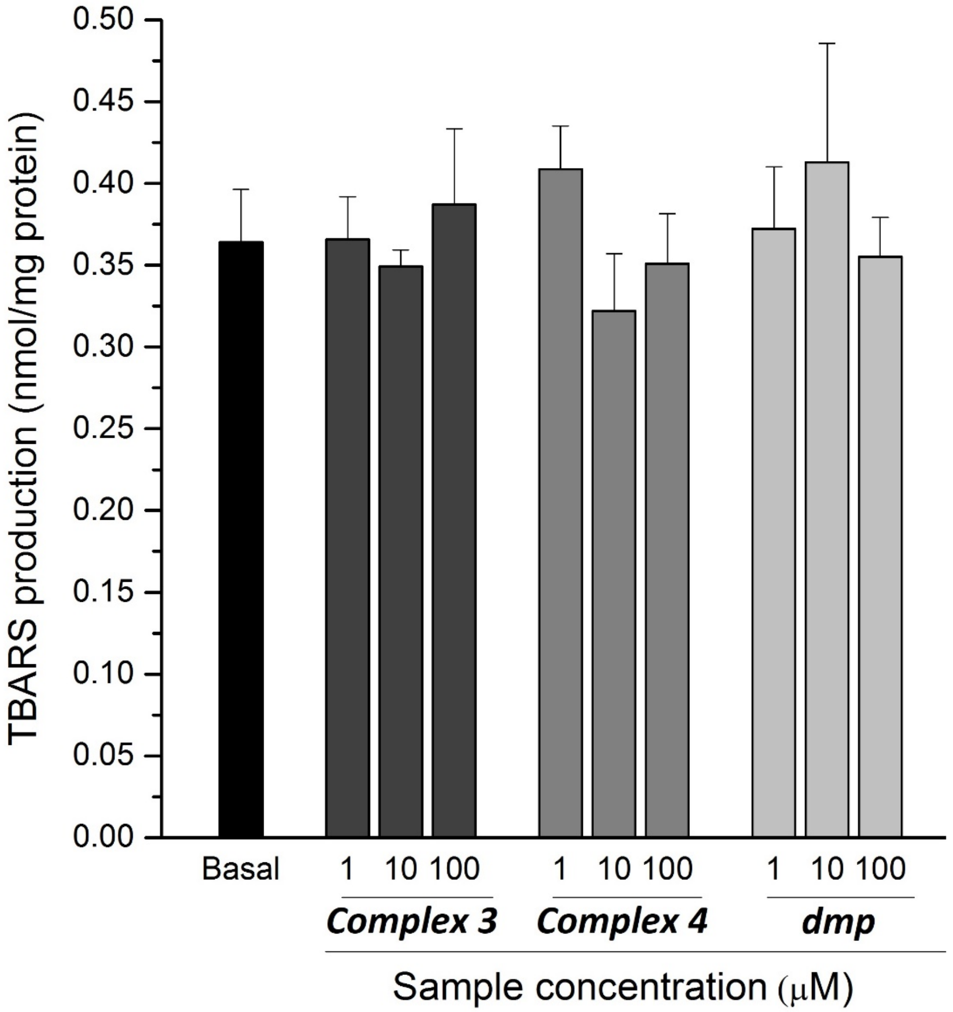

Fig. S20. Effect of concentration on the production of TBARS in the presence of compounds **3**, **4**, and dmp ligand.

Table S6. Relative migration ratio % of **3** and **4** against cancer lines at different times.

**Cell line**	**Time**	**Treatments**			
		**Control**	**Compound 3 (5µM)**	**Compound 4 (5µM)**	**MTX (0.5µM)**
**MCF7**	24h	53.4 ±2.6	22.29±2.2	34.3±2.4	33.5±1.6
	48h	84.7±2.9	35.2±3.6	49.0±3.6	60.5±1.5
**PC-3**	24h	48.7±5.0	23.1±2.0	37.5±3.1	24.2±1.6
	48h	98.6±0.9	37.2±4.5	58.1±4.8	33.7±6.2
**SKLU-1**	24h	23.4±2.0	20.2±2.3	15.1±2.0	22.9±2.4
	48h	41.5±2.1	21.4±2.4	24.1±3.3	35.7±3.1
